# UCHL3 depletion inhibits gastric cancer progression and enhances palbociclib sensitivity by regulating the AKT/CCND1 signaling axis via ENO1 ubiquitination

**DOI:** 10.1038/s41419-025-08153-3

**Published:** 2025-11-21

**Authors:** Weiqi Liu, Ling Zhou, Yi Le, Yan He, Juanjuan Zhou, Hongjiao Zhang, Jinbo Zhan, Tingting Hu, Jingru Wang, Yun Lin, Haiming Yu, Jianping Xiong, Ziling Fang, Xiaojun Xiang

**Affiliations:** Department of Oncology, The First Affiliated Hospital, Jiangxi Medical College, Nanchang University, Nanchang, Jiangxi Province P.R. China

**Keywords:** Gastric cancer, Tumour biomarkers

## Abstract

Ubiquitin carboxyl-terminal hydrolase L3 (UCHL3) is a deubiquitinating enzyme involved in various cancers, yet its role in gastric cancer (GC) requires further exploration. This study primarily investigates the expression, function, and mechanisms of UCHL3 in GC. Clinical samples and bioinformatics analysis indicated that UCHL3 is overexpressed in GC tissues compared to adjacent normal tissues, with higher expression levels correlating with worse prognosis. Functional assays demonstrated that UCHL3 promotes GC cell proliferation, invasion, and migration, accelerates cell cycle progression, and induces epithelial–mesenchymal transition (EMT). In vivo studies using a cell line-derived xenograft (CDX) model confirmed that UCHL3 enhances GC proliferation, and its therapeutic potential was validated in patient-derived xenografts (PDX). Mechanistically, transcriptomic analysis and validation experiments identified the AKT/CCND1 signaling pathway as a key mediator of UCHL3-driven GC progression. Co-immunoprecipitation (Co-IP) and liquid chromatography-mass spectrometry identified potential UCHL3-binding proteins, notably the AKT activator ENO1. Molecular docking simulations, Co-IP, and GST-pull down assays further confirmed the interaction, mapping the binding regions between UCHL3 (AA 179-230) and ENO1 (AA 140-434). Cycloheximide (CHX) and in vivo ubiquitination assays demonstrated that UCHL3 deubiquitinates and stabilizes ENO1, thus extending its half-life, while UCHL3 inhibition produced the opposite effect. A C95A point mutation significantly impaired UCHL3’s deubiquitination function on ENO1. Further studies revealed UCHL3 removes K48-linked polyubiquitin chains from ENO1 at lysine 92, activating the AKT/CCND1 signaling pathway. In addition, the small-molecule inhibitor TCID, specific for UCHL3, inhibited this deubiquitination, counteracting pro-tumorigenic effects. In vitro and in vivo experiments demonstrated that TCID increased the sensitivity of GC cells to CDK4/6 inhibitors palbociclib. These findings suggest that UCHL3 contributes to GC progression and represents a promising therapeutic target for GC treatment.

## Introduction

Gastric cancer (GC) is a prevalent malignancy in the digestive system, exhibiting persistently high incidence and mortality rates [1]. The unlimited proliferation and metastasis of tumor cells are key features of malignancies [2], significantly contributing to poor treatment outcomes among GC patients. Consequently, exploring the molecular mechanisms driving GC proliferation and invasion, and identifying valuable biomarkers or therapeutic targets within the intricate regulatory networks, is of utmost importance.

Ubiquitin carboxyl-terminal hydrolase L3 (UCHL3), a member of the UCH family, acts as a deubiquitinating enzyme within the ubiquitin-proteasome system [3]. Comprising 230 amino acids, UCHL3 contains a crucial UCH domain that facilitates specific substrate binding, enabling its vital biological function as a deubiquitinase [4]. In recent decades, the expression and regulatory mechanisms of UCHL3 in solid tumors have garnered widespread attention [5]. Previous studies have indicated a significant upregulation of UCHL3 expression in several cancers, such as hepatocellular carcinoma, breast cancer, and colorectal cancer, correlating with poor prognosis and suggesting its potential as a therapeutic target [6–8]. Nevertheless, the specific role and the underlying mechanism of UCHL3 in GC still require further exploration.

CDK4/6 are crucial cyclin-dependent protein kinases that form complexes with Cyclin D to drive the cell cycle [9]. Several inhibitors, such as palbociclib, ribociclib, and abemaciclib, have been developed to block CDK4/6 activation [10]. These inhibitors have shown therapeutic potential across various cancer types but are primarily used for treating HR + , HER2- advanced breast cancer [11, 12]. Numerous clinical studies are exploring the efficacy of CDK4/6 inhibitors in advanced solid tumors, including GC [11]. Notably, a phase I clinical study (NCT03480256) evaluated the efficacy and safety of dalpiciclib combined with pyrotinib in HER2+ advanced GC patients who had failed prior systemic therapy, showing promising results and offering a new treatment option [13]. 4,5,6,7-Tetrachloroindan-1,3-dione (TCID), a small-molecule inhibitor of UCHL3 [14], has not yet been tested in combination with CDK4/6 inhibitors for GC, but its potential benefits are worth exploring.

In this study, we found that UCHL3 is highly expressed in GC tissues and closely associated with poor prognosis and adverse pathological features through bioinformatics analysis and GC tissue samples. Overexpression of UCHL3 promotes GC proliferation, invasion, and migration. More importantly, we discovered that UCHL3 enhances the deubiquitination of ENO1 linked by K48 ubiquitin, thereby stabilizing the ENO1 protein and leading to the activation of the AKT/CCND1 signaling axis. In addition, the small-molecule inhibitor TCID increased the sensitivity of GC cells to CDK4/6 inhibitors, highlighting its potential as a therapeutic strategy.

## Materials and methods

### Tissue specimens and ethics statement

Paraffin-embedded tissue samples were collected from 127 GC patients treated at the First Affiliated Hospital of Nanchang University, between January 2016 and September 2017. Fresh GC tissue samples were obtained from the surgical department of our hospital between June 2021 and June 2022. These patients underwent surgery without prior anti-cancer therapy. Ethical approval for this study was obtained from the Ethics Committee of the First Affiliated Hospital of Nanchang University, and informed consent was obtained from all patients involved.

### Immunohistochemistry (IHC) assays

The GC tissue and xenograft tumor tissue samples were stained immunohistochemically using the previously described method [15]. Scoring was based on staining intensity (1–4) and the percentage of positively stained cells, with staining intensity ranging from 0 (no staining) to 3 (strong staining), and the percentage of positively stained cells categorized into four groups: ≤25%, 26–50%, 51–75%, and >75%. The immunohistochemical score for each sample was calculated by multiplying the staining intensity by the percentage of positively stained cells. A score of less than 6 indicated low UCHL3 expression, while a score of 6 or higher indicated high UCHL3 expression.

### Cell culture and transfection

Human GC cell lines MKN-45, AGS, HGC-27, MKN-28, NCI-N87, human gastric mucosal epithelial cell line GES-1, and human embryonic kidney cell line HEK-293T were acquired from the Cell Bank of the Chinese Academy of Sciences in Shanghai, China. SNU-719 cell line was sourced from the Korean Cell Line Bank and generously donated through Shanghai Fudan University. The majority of GC cells were cultured in RPMI-1640 medium supplemented with 10% fetal bovine serum (FBS), while HEK-293T and GES-1 cells were cultured in DMEM medium containing 10% FBS. All cells were authenticated using short tandem repeat (STR) profiling, confirmed to be mycoplasma-free, and maintained in a humidified incubator at 37 °C with 5% CO_2_.

In cell transfection experiments, transfection reagents, comprising Opti-MEM (31985070, Thermo Fisher Scientific, USA), TurboFect Reagent (FERR0534, Thermo Fisher Scientific, USA), and either overexpression plasmids or siRNA, were prepared and mixed according to the manufacturer’s instructions. The resulting mixture was then added to culture dishes containing cells at 30–50% confluency. After incubation for 36–48 h under appropriate conditions, the transfected cells were used for subsequent experiments. The overexpression plasmids and siRNA utilized in this study were constructed by Suzhou Gima Biotechnology Co., Ltd. Further details can be found in Supplementary Table S1.

### Protein extraction and western blotting analysis

Protein extraction from GC tissue, cells, and xenograft tumor tissues was conducted according to previously described methods [16]. Briefly, tissues were washed, minced, and lysed in RIPA lysis solution with protease inhibitors and phosphatase inhibitors. Detection of the target protein was performed using Western blotting. Protein samples were denatured with loading buffer at high temperature, followed by separation by SDS-PAGE, transfer to a membrane, blocking, primary antibody incubation, washing, secondary antibody incubation, and detection. Protein bands were visualized to analyze protein expression levels or modifications. All primary antibody information is listed in Supplementary Table 2.

### RNA extraction, RNA-seq, and quantitative real-time PCR (qRT‒PCR) assays

Total RNA was extracted using TRIzol reagent (9108, Takara, Tokyo, Japan) according to the manufacturer’s instructions. The extracted RNA was then reverse transcribed into cDNA using a reverse transcription kit (RR047A, Takara, Tokyo, Japan). qRT-PCR was performed using specific primers and SYBR Green Master Mix (RR820A, Takara, Tokyo, Japan) on a real-time PCR system (CFX384, Bio-Rad, Munich, Germany). The cycling conditions included an initial denaturation step, followed by amplification cycles, and a final extension step. Gene expression levels were quantified using the 2^−ΔΔCt^ method, normalized to an internal control, and presented relative to a reference sample. The primer information for this experiment is provided in Supplementary Table 3.

### Co-immunoprecipitation (Co-IP) assays

For endogenous Co-IP assays, total cellular protein was extracted, followed by incubation with specific antibodies overnight. Antibody-protein complexes were then captured using ProteinA/G beads, washed to remove nonspecifically bound proteins, eluted, and subjected to SDS-PAGE for analysis. For exogenous Co-IP experiments, Flag-UCHL3 and HA-ENO1 overexpression plasmids were transfected into HEK-293T cells. Subsequently, Anti-DYKDDDDK Magnetic beads (M185-3L, MBL International, Woburn, MA, USA) or Anti-HA Magnetic beads (HY-K0201, MCE, Shanghai, China) were used for pulldown, followed by the same steps as in endogenous Co-IP assays.

### GST-pull down assays

Purified His-UCHL3 (Ag32925) and GST-ENO1 (Ag1692) fusion proteins were purchased from Proteintech (Wuhan, China). For the GST pull-down assay, His-UCHL3 and GST-ENO1 fusion proteins were incubated in binding buffer at 4 °C overnight, followed by incubation with Anti-GST Magnetic Beads for an additional 4 h. After multiple washes with the binding buffer, the pull-down samples were separated by SDS-PAGE and visualized using Coomassie Brilliant Blue staining as previously reported [17].

### Cell counting kit-8 (CCK-8) and colony formation assays

Cell Counting Kit-8 (CCK-8) assays were conducted to assess cell viability. Transfected cells were seeded into 96-well plates at a density of 1000 cells per well and cultured for 24, 48, 72, and 96 h at 37 °C in a humidified atmosphere with 5% CO_2_. CCK-8 solution was added to each well according to the manufacturer’s instructions, and the plates were further incubated for 1.5 h. Absorbance was measured at 450 nm using a microplate reader.

For colony formation assays, transfected cells were seeded into six-well plates at a density of 1000 cells per well and cultured for ~14 days. Cells were transfected with the corresponding SiRNA every 3 days to maintain the knockdown efficiency. After incubation, cell colonies were fixed with paraformaldehyde, stained with crystal violet, and colonies containing at least 50 cells were counted.

### Wound healing and transwell assays

Wound healing and Transwell assays were performed to evaluate cell invasion or migration. For the wound healing assay, cells were grown to confluence in six-well plates, and a scratch was made using a sterile pipette tip. Images were captured at 0, 24, and 48 h post-scratching to assess wound closure. In addition, transfected cells were seeded onto the upper chamber of Transwell inserts coated with Matrigel for invasion assays. The lower chamber contained complete culture medium. After a specified incubation period, invaded cells were fixed, stained, and counted under a microscope. Experiments were conducted at least three times for reliability.

### Immunofluorescence staining assays

Cells were seeded onto glass coverslips in six-well plates and allowed to adhere overnight. After fixation with 4% paraformaldehyde and permeabilization with 0.25% Triton X-100, cells were blocked with 5% bovine serum albumin (BSA) for 1 h at room temperature. Subsequently, two different primary antibodies were applied and incubated overnight at 4 °C. Following thorough washing, cells were incubated with corresponding fluorophore-conjugated secondary antibodies for 1 h at room temperature. After washing off the excess secondary antibody, the cells were stained with DAPI for nuclear visualization. Images were then acquired using a confocal fluorescence inverted microscope (Stellaris 5, Leica Microsystems, Nussloch, Germany).

### Cycloheximide assays and in vivo ubiquitination analysis

For Cycloheximide assays, GC cells transfected with either UCHL3 overexpression or knockdown constructs were treated with 100 μM cycloheximide (CHX) to suppress protein translation. Samples were collected at specific time points (0, 3, 6, 9, and 12 h for the UCHL3 overexpression and control groups; 0, 2, 4, and 6 h for the UCHL3 knockdown and control groups) for protein extraction and subsequent western blotting analysis to assess protein degradation kinetics.

For in vivo ubiquitination analysis, HEK-293T cells were transfected with His-Ub, HA-ENO1, and Flag-UCHL3 plasmids as required. After 48 h of transfection, cells were collected. Six hours before cell collection, MG132 (a proteasome inhibitor) was added to the cell culture dishes. Subsequently, cells were lysed, and HA-ENO1 proteins were immunoprecipitated using anti-HA agarose beads. The Co-IP assays were then subjected to western blotting analysis to assess the ubiquitination level of ENO1.

### Animal models

The xenograft tumor model was established using MKN-28 and HGC-27 cells. First, stable overexpression or knockdown of UCHL3 was achieved in GC cells using lentiviral vectors. Subsequently, cultured cancer cells were harvested and resuspended in an appropriate medium. Approximately 5 × 10^6^ cells were then subcutaneously injected into the right axilla of nude mice, and tumor growth was regularly monitored. Tumor volume was calculated using the formula: volume = (length × width^2^)/2. After approximately 28 days of growth, mice were euthanized, and tumors were excised for further analysis.

To explore UCHL3’s therapeutic potential, GC patient samples were fragmented and implanted into nude mice to establish patient-derived xenografts (PDX) models. PDX models with passages ranging from 3 to 10 were utilized. Upon reaching a tumor volume of 50–100 mm^3^, intratumoral injections of lentiviral vectors LV-NC and LV-shUCHL3 were administered at a concentration of 1 × 10^8^ TU per injection per mouse every three days, and tumor growth was monitored. All animal experiments were approved by the Institutional Animal Care and Use Committee of the First Affiliated Hospital of Nanchang University.

### Bioinformatics analysis

In this study, the TCGA, GEO, and GTEx databases were utilized to compare the expression differences of UCHL3 in GC tissues and adjacent non-cancerous tissues. DNA mutations in various deubiquitinating enzymes were analyzed using the cBioPortal website. Molecular docking simulations and site predictions were performed using Schrödinger software.

### Statistical analysis

The statistical analysis was performed using SPSS Statistics (Version 27). Kaplan–Meier method was utilized to generate survival analysis curves for patients with different UCHL3 expressions, and comparisons were made using Log-rank test. Experimental data are presented as mean ± standard error of the mean (SEM), and Student’s two-tailed *t* test or one-way analysis of variance (ANOVA) was employed to compare differences among groups, with significance level set at *P* < 0.05.

## Results

### UCHL3 is overexpressed in GC and correlates with poor prognosis

**Fig. 1:**
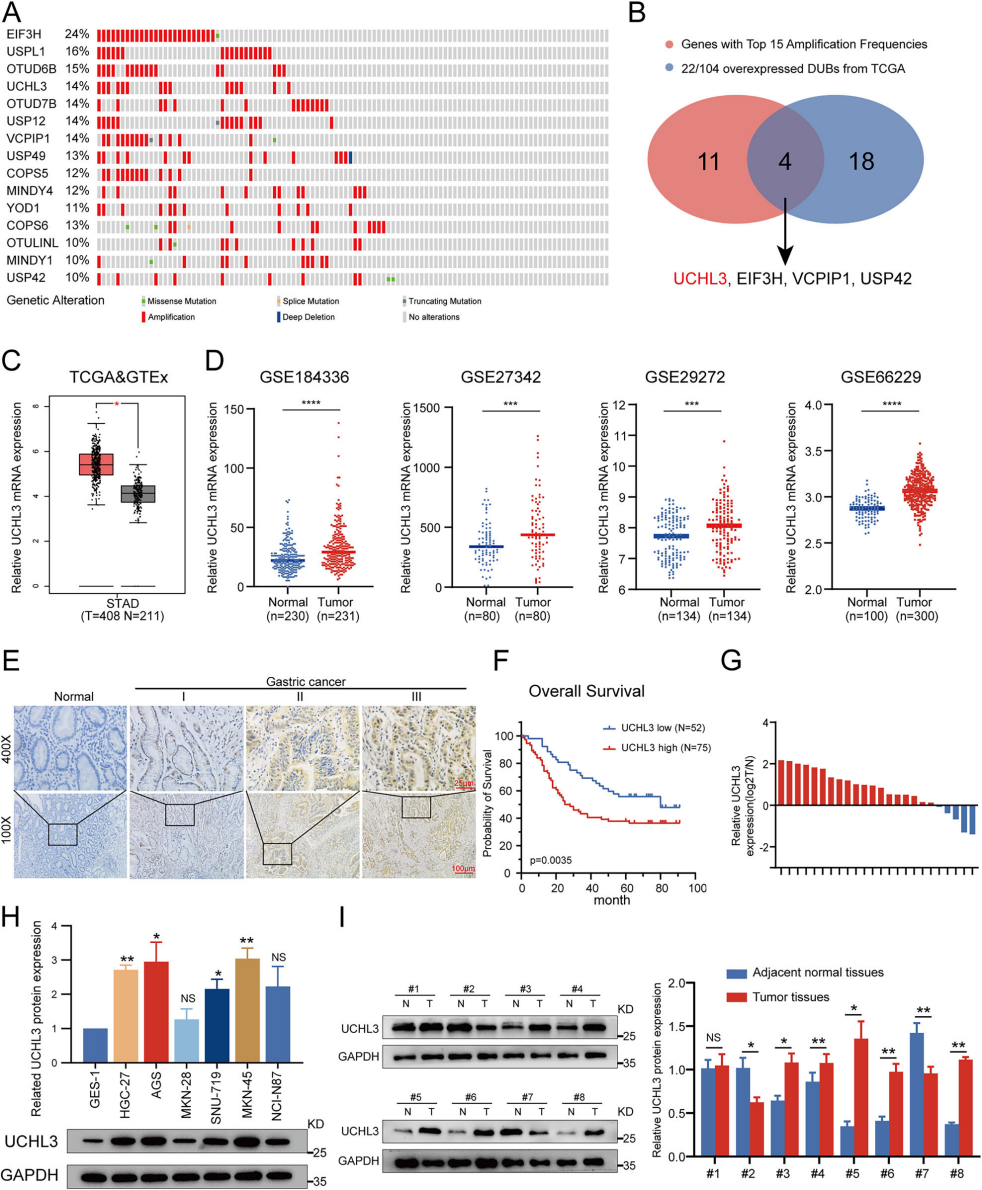
UCHL3 is overexpressed in GC and correlates with poor prognosis. **A** Exploration of gene mutations in deubiquitinating enzymes in gastric cancer (GC) using the cBioPortal website (https://www.cbioportal.org/, dataset: OncoSG, 2018), highlighting the top 15 genes with the highest amplification rates. **B** Intersection of significantly differentially expressed deubiquitinating enzyme genes in TCGA GC data with the top 15 genes exhibiting significant amplification mutations. **C** Differential expression of UCHL3 in GC tissues and adjacent tissues from the TCGA and GTEx databases using the GEPIA website (http://gepia.cancer-pku.cn/). **D** Analysis of UCHL3 expression in GEO datasets GSE184336, GSE27342, GSE29272, and GSE66229 (https://www.ncbi.nlm.nih.gov/geo/). **E** Representative IHC images of UCHL3 in GC tissues or adjacent tissues. **F** Survival analysis of the 127 GC patients based on IHC scores of UCHL3. **G** RT-qPCR analysis of UCHL3 expression in 20 pairs of GC tissues and matched adjacent normal tissues, presented as log2 (Tumor/Normal). **H**, **I** Representative Western blotting images and quantitative analysis of UCHL3 expression in GC cell lines and the gastric mucosal cell line GES-1 (**H**), and in eight pairs of GC tissues and matched adjacent normal tissues (**I**). Student’s *t* test: *P < 0.05, **P < 0.01, ***P < 0.001, ****P < 0.0001.

Deubiquitinating enzymes play a crucial role in tumorigenesis [18], however, their involvement in GC requires further investigation. To address this, we assessed gene mutation patterns across 110 deubiquitinating enzymes in the latest dataset “Gastric Cancer (OncoSG, 2018)” from the cBioPortal website (https://www.cbioportal.org/), and found amplification mutations to be predominant (Supplementary Fig. S1A). In addition, we highlighted the top 15 genes in Fig. 1A. Further examination of mRNA expression profiles from TCGA data revealed significant differences in 22 deubiquitinating enzymes. By taking the intersection, we identified UCHL3, EIF3H, VCPIP1, and USP42 as potential candidates (Fig. 1B). Among these, UCHL3 showed the most significant differential expression in 24 paired GC specimens (Supplementary Fig. S1B), prompting us to focus on it for further study. Both the TCGA+GTEx databases and several large-scale GEO datasets consistently demonstrated higher UCHL3 expression in GC tissues compared to adjacent normal tissues (Fig. 1C, D). Subsequently, we validated these findings through IHC staining of paraffin-embedded samples from 127 GC patients. The results confirmed higher UCHL3 expression in tumor tissues, with significant positive correlations to TNM staging (Fig. 1E). Patients with high UCHL3 expression had significantly poorer prognosis (*P* = 0.0035) than those with low expression (Fig. 1F). Chi-square tests further revealed that elevated UCHL3 expression was closely associated with tumor size (*P* = 0.043), TNM staging (*P* = 0.028), and lymph node metastasis (*P* = 0.029) (Table 1). Immunoblotting analysis and RT-qPCR confirmed increased UCHL3 expression in the majority of GC tissues and cells compared to normal controls (Fig. 1G–I). Overall, our findings indicate that UCHL3 overexpression in GC is closely linked to adverse patient prognosis.

**Table 1:** Association of UCHL3 levels with clinical and pathological features of GC patients.

Variables	*N*	Tumor UCHL3 expression		*P* value
		UCHL3^High^ (%)	UCHL3^Low^ (%)	
Age (years)				
<65	91	56 (61.5)	35 (38.5)	0.366
≥65	36	19 (52.8)	17 (47.2)	
Gender				
Male	89	53 (59.6)	36 (40.4)	0.862
Female	38	22 (57.9)	16 (42.1)	
Tumor size (cm)				
<5	77	40 (51.9)	37 (48.1)	**0.043**
≥5	50	35 (70.0)	15 (30.0)	
Differentiation				
Well or moderately poor	36	20 (55.6)	16 (44.4)	0.614
	91	55 (60.4)	36 (39.6)	
TNM stage				
I-II	29	12 (41.4)	17 (58.6)	**0.028**
III-IV	98	63 (64.3)	35 (35.7)	
Depth of invasion				
T1-T3	11	6 (54.5)	5 (45.5)	0.750
T4	116	69 (59.1)	47 (40.5)	
Tumor location				
Proximal	44	30 (68.2)	14 (31.8)	0.128
Distal	83	45 (54.2)	38 (45.8)	
Lauren classification				
Intestinal type	74	47 (63.5)	27 (36.5)	0.227
Diffuse type	43	28 (52.8)	25 (47.2)	
Lymph node metastasis				
N0-1	40	18 (45.0)	22 (55.0)	**0.029**
N2-3	87	57 (65.5)	30 (34.5)	

*P* values determined using the χ^2^ test.

### UCHL3 promotes GC cell proliferation, invasion, migration, accelerates cell cycle progression, and induces epithelial–mesenchymal transition (EMT)

**Fig. 2:**
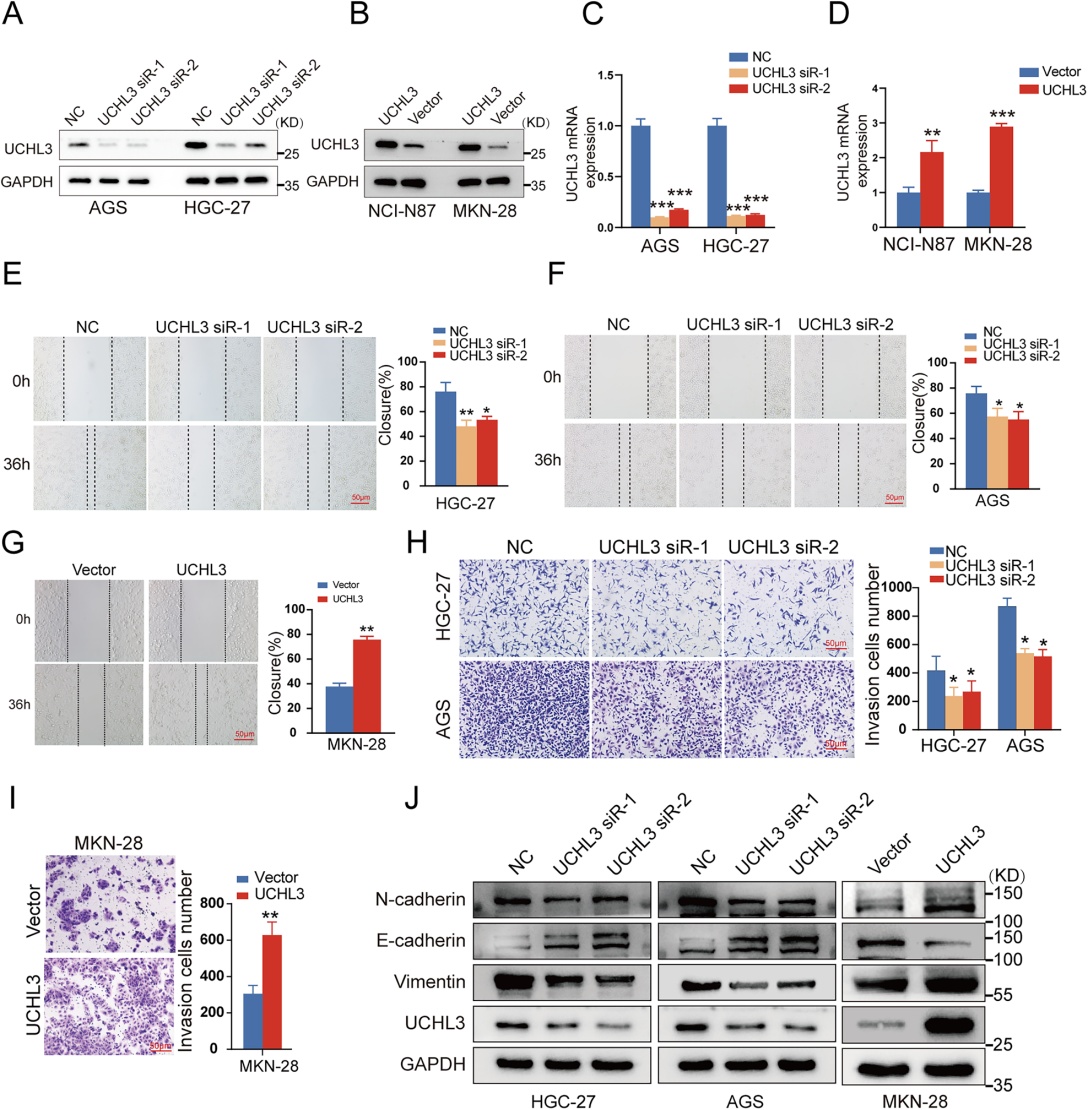
UCHL3 enhances invasion, migration, and EMT in GC cells. GC cells were transfected with either UCHL3 siRNA or UCHL3 overexpression plasmid, and subsequent experiments were performed 48 h post-transfection. UCHL3 elevation was performed in HGC-27 and AGS cells, while UCHL3 suppression was conducted in MKN-28 cells. **A**–**D** Western blotting and RT-qPCR to assess the knockdown efficiency of UCHL3 siRNA or plasmid. **E**–**G** Wound healing assay used to analyze the effects of UCHL3 upregulation or silencing on GC cell migration ability. **H**, **I** Representative images and quantitative results of the Transwell invasion assay assessing the impact of UCHL3 elevation or suppression on cell invasion ability. **J** Western blotting image depicting the protein expression of E-cadherin, N-cadherin, and Vimentin, used to evaluate the impact of UCHL3 expression on EMT in GC cells. All experiments were repeated at least three times. Student’s *t* test: *P < 0.05, **P < 0.01, ***P < 0.001.

**Fig. 3:**
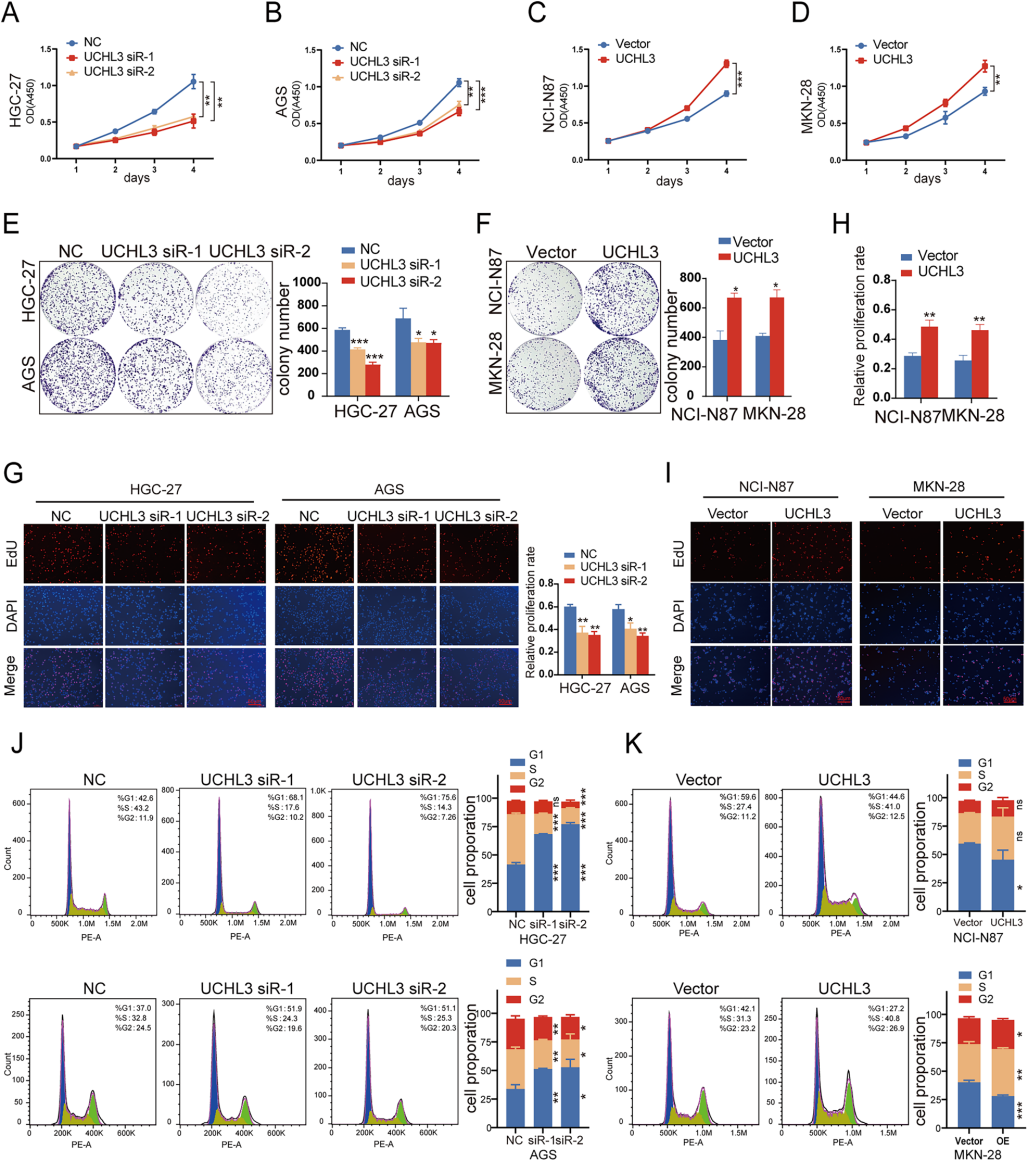
UCHL3 promotes GC cell proliferation and accelerates cell cycle progression. **A**–**H** Impact of UCHL3 knockdown or upregulation on the proliferation capacity of GC cells, respectively, assessed by the CCK-8 assay (**A**–**D**); colony formation assays (**E**, **F**)； and EdU assays (**G**–**I**). **J**, **K** Representative flow cytometry plots display cell cycle distribution in transfected GC cells, along with quantitative analysis of cell population percentages in G1, S, and G2/M phases. All experiments were repeated at least three times. Student’s *t* test: *P < 0.05, **P < 0.01, ***P < 0.001.

To delve deeper into UCHL3’s involvement in GC, we modulated its expression in GC cells using UCHL3-specific siRNA or overexpression plasmids. Subsequently, we validated the transfection efficiency through western blotting and RT-qPCR assays before conducting a series of cellular functional assays (Fig. 2A–D). Wound healing and transwell invasion assays indicated that UCHL3 knockdown significantly reduced the migratory and invasive capabilities of GC cells (Fig. 2E, F, H). In contrast, elevated UCHL3 expression enhanced these properties in MKN-28 cells, as evidenced by increased scratch closure and invasion rates (Fig. 2G, I). Moreover, alterations in epithelial–mesenchymal transition (EMT) were evident, characterized by heightened expression of E-cadherin and diminished levels of N-cadherin and Vimentin in the UCHL3 knockdown group, whereas in the overexpression group, E-cadherin expression decreased while N-cadherin and Vimentin expression increased (Fig. 2J). In addition, CCK-8 assays, colony formation assays, and EdU assays consistently showed that UCHL3 knockdown suppressed the proliferation abilities of HGC-27 and AGS cells, whereas UCHL3 overexpression exerted the opposite effect (Fig. 3A–I). In addition, flow cytometry analysis further revealed that UCHL3 depletion induced G1/S phase arrest, marked by an increased G0/G1 population and decreased S and G2/M fractions in HGC-27 and AGS cells (Fig. 3J). Conversely, overexpression of UCHL3 promoted G1/S transition in MKN-28 and NCI-N87 cells (Fig. 3K). In summary, UCHL3 enhances GC cell proliferation, migration, invasion, and EMT, primarily through promoting cell cycle progression and EMT, underscoring its pivotal role in GC progression.

### UCHL3 regulates the AKT/CCND1 signaling pathway

**Fig. 4:**
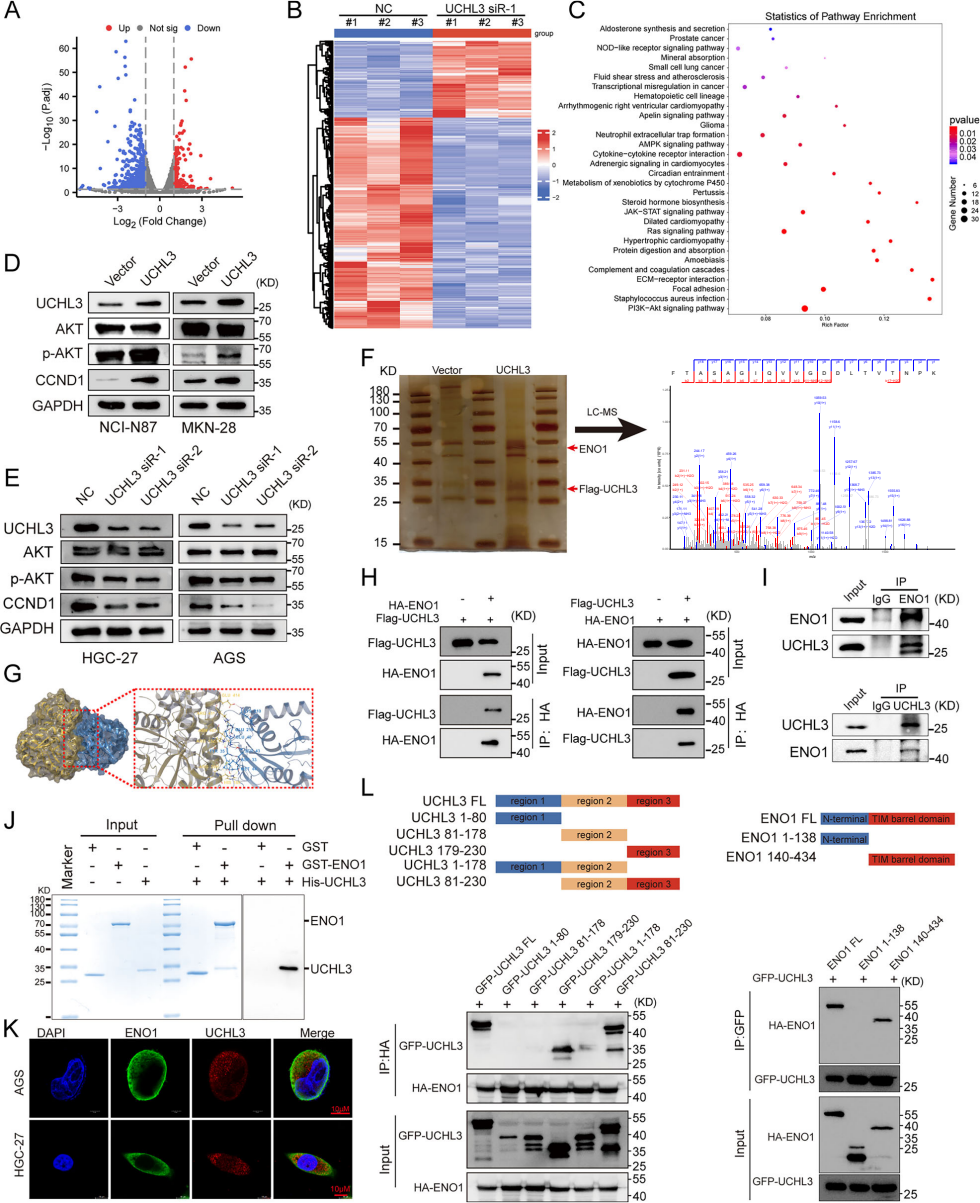
UCHL3 regulates the AKT/CCND1 axis and interacts with the AKT activator ENO1. **A**–**C** Transcriptome sequencing was performed in HGC-27 cells with UCHL3 knockdown. Volcano plot of gene expression in UCHL3 knockdown vs. control group (**A**); Heatmap of differentially expressed genes (**B**); KEGG analysis of differentially expressed genes (**C**). **D**, **E** Western blot analysis of changes in total AKT, phosphorylated AKT (p-AKT, S473), and downstream target gene CCND1 after UCHL3 modulation. **F** Silver-stained SDS-PAGE image of Flag-immunoprecipitated proteins isolated from HGC-27 cells transfected with Flag-UCHL3 or empty vector and peptide of ENO1 identified by Mass spectrometry (MS)**. G** Molecular docking of UCHL3 with ENO1 using Schrödinger software. **H** Exogenous co-immunoprecipitation (Co-IP) assay to detect the interaction between Flag-UCHL3 and HA-ENO1. **I** Endogenous Co-IP assay to detect the interaction between UCHL3 and ENO1 in HGC-27 cells. **J** GST-pull down assay detecting the direct binding between UCHL3 and ENO1. **K** Dual-immunofluorescence assay to detect the localization of UCHL3 and ENO1 in AGS and HGC-27 cells. **L** Schematic representation of truncated plasmid construction, and immunoprecipitation assay detecting the binding region between UCHL3 and ENO1.

To further investigate how UCHL3 drives GC progression, we silenced UCHL3 in GC cells and performed transcriptome sequencing. This analysis identified 991 differentially expressed genes, with 331 upregulated and 660 downregulated (Fig. 4A, B). KEGG pathway analysis revealed significant enrichment of the differentially expressed genes in the PI3K/AKT pathway (Fig. 4C), with CCND1, a key downstream target of AKT, exhibiting marked differential expression (Supplementary Fig. S2A). In addition, TCGA GC data revealed a positive correlation between UCHL3 and CCND1 expression (Supplementary Fig. S2B). Next, we performed experiments to validate UCHL3’s regulation of the AKT/CCND1 signaling axis. Western blotting analysis showed that UCHL3 knockdown decreased phosphorylated AKT (S473) and CCND1 protein expression, while overexpression had the opposite effect, with no impact on total AKT levels (Fig. 4D, E). RT-qPCR confirmed a positive correlation between UCHL3 and CCND1 expression (Supplementary Fig. S2C–F). Although AKT-mediated regulation of CCND1 is well-documented [19–21], our inhibitor experiments using MK-2206 demonstrated that UCHL3 activates CCND1 through AKT. Treatment with MK-2206 completely reversed the UCHL3 overexpression-induced CCND1 upregulation (Supplementary Fig. S2G, H), establishing AKT as the mediator in this regulatory cascade. These findings suggest that UCHL3 promotes GC progression by modulating the AKT/CCND1 signaling pathway.

### UCHL3 interacts with ENO1

Using immunoprecipitation and mass spectrometry, we identified potential interacting proteins with UCHL3, including ENO1 (Fig. 4F and Supplementary Table 4), which has been reported as an activator of AKT [22–25]. Given ENO1’s role in activating AKT signaling, we hypothesize that UCHL3 orchestrated the AKT/CCND1 axis through ENO1. Thus, molecular docking was performed to further explore the interaction between UCHL3 and ENO1, revealing a strong interaction with a binding energy of -647.804 kcal/mol (Fig. 4G, enlarged figure shown in Supplementary Fig. S2I). Furthermore, we confirmed the interaction between UCHL3 and ENO1 by transfecting Flag-UCHL3 and HA-ENO1 plasmids into 293 T cells and performing exogenous Co-IP experiments, which demonstrated their interaction (Fig. 4H). This interaction was also supported by endogenous Co-IP results in GC cells (Fig. 4I). Moreover, GST-pull down experiments using purified His-UCHL3 and GST-ENO1 fusion proteins confirmed a direct interaction between the two proteins (Fig. 4J). Furthermore, Immunofluorescence staining of HGC-27 and AGS cells revealed cytoplasmic colocalization of UCHL3 and ENO1, providing further evidence of their interaction in situ (Fig. 4K). To further explore their binding regions, we constructed a series of truncated plasmids for UCHL3 and ENO1, and found that the UCHL3 (179-230 AA) fragment interacted with the ENO1 (140-434 AA) fragment through Co-IP assays (Fig. 4L).

### UCHL3 deubiquitinates and stabilizes ENO1

**Fig. 5:**
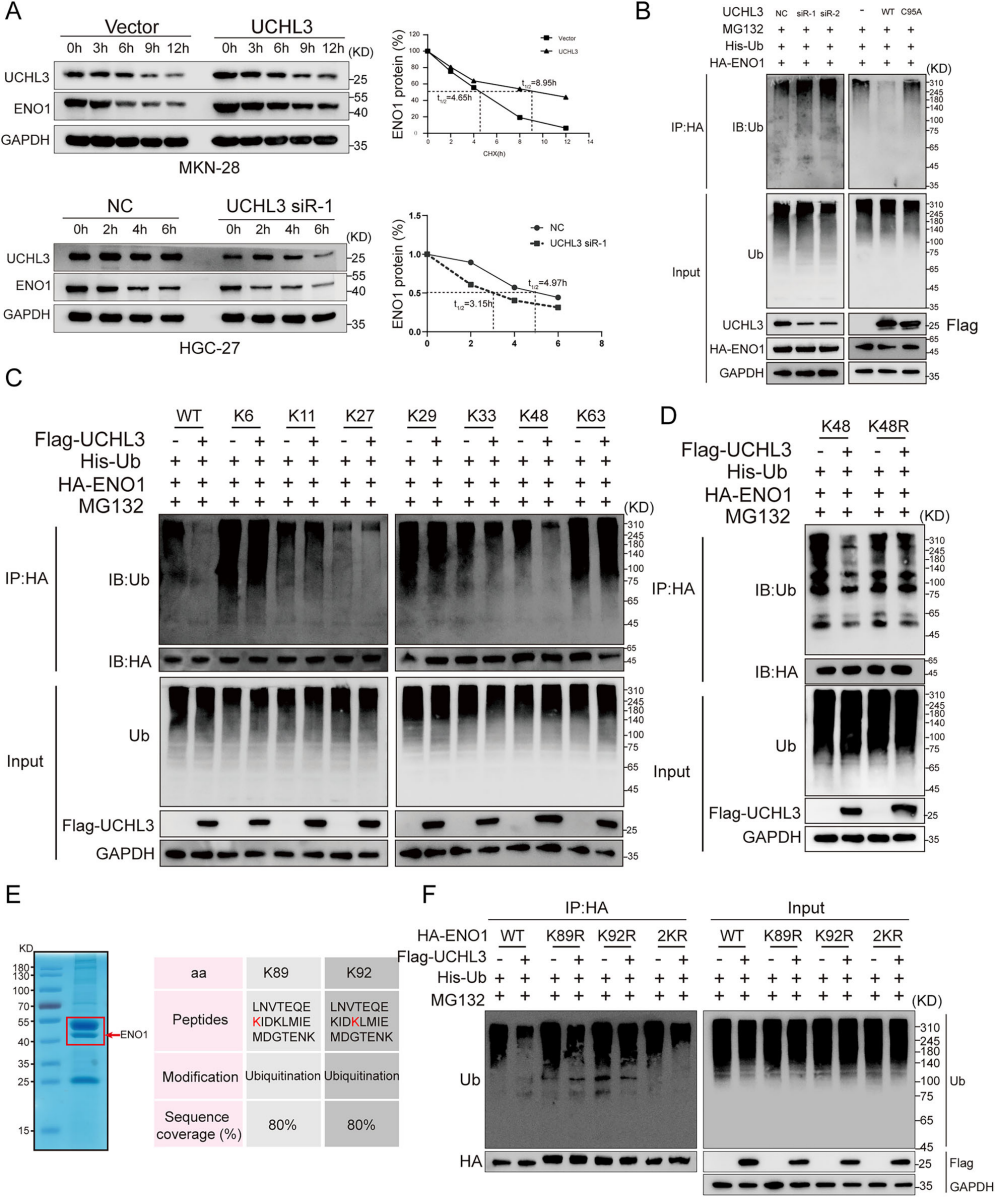
UCHL3 deubiquitinates and stabilizes ENO1. **A** Cycloheximide (CHX, 100 μM) treatment of transfected GC cells, with proteins collected at the indicated time points to assess ENO1 protein levels. The protein degradation curve was plotted, and the half-life of ENO1 was calculated. **B** In vivo ubiquitination assay examining the impact of UCHL3 expression and its active site mutations on the ubiquitination levels of ENO1. **C** Mutant plasmids for K6, K11, K27, K29, K33, K48, and K63 were constructed, and in vivo ubiquitination assays were used to determine the deubiquitination patterns of ENO1 by UCHL3. **D** K48 and K48R plasmids were introduced to further confirm the deubiquitination pattern of ENO1 by UCHL3 via in vivo ubiquitination assays. **E** Immunoprecipitation was used to collect ENO1 protein for mass spectrometry(MS) analysis to identify ubiquitination sites. **F** Based on the results of MS, plasmids with ENO1 mutations at lysine 89, 92, and both sites were constructed. In vivo ubiquitination assays were then performed to determine the specific deubiquitination sites of ENO1 by UCHL3.

UCHL3, a deubiquitinating enzyme, plays a crucial biological role by specifically binding to target proteins and removing their ubiquitination chain [26, 27]. Building on our findings of a strong interaction between UCHL3 and ENO1, we conducted further experiments to elucidate how UCHL3 affects ENO1. Initially, we observed that manipulating UCHL3 expression in GC cell lines correspondingly altered ENO1 protein levels. Overexpression of UCHL3 increased ENO1 levels, while knockdown of UCHL3 reduced them (Supplementary Fig. S3A, B). In addition, cycloheximide (CHX) protein half-life assays demonstrated that UCHL3 overexpression prolonged ENO1’s half-life, whereas UCHL3 silencing shortened it (Fig. 5A), indicating that UCHL3 stabilizes ENO1. In vivo ubiquitination experiments were performed to validate whether UCHL3 stabilizes ENO1 protein expression through deubiquitination. As shown in Fig. 4B, reducing UCHL3 expression increased ENO1 ubiquitination levels, while enhancing UCHL3 expression reduced it. The 95th amino acid has been reported as a critical site for UCHL3’s critical function [28]. We introduced a C95A mutation, which abolished UCHL3’s deubiquitination effect on ENO1 (Fig. 5B). Furthermore, a dose-dependent correlation between UCHL3 expression and ENO1 deubiquitination was observed. Specifically, higher UCHL3 expression levels were associated with reduced ENO1 ubiquitination, a trend consistently observed in both two GC cell lines (Supplementary Fig. S3C, D). Given that different types of ubiquitination serve distinct functions, with K48 linkage being the most common among all ubiquitin chains, accounting for approximately 50% of all linkages and closely associated with proteasomal degradation [29, 30], Therefore, mutant plasmids for K6, K11, K27, K29, K33, K48, and K63 were constructed for in vivo ubiquitination experiments to identify the specific type of ubiquitination chain that UCHL3 targets to stabilize ENO1. As shown in Fig. 5C, we found that UCHL3 significantly removes K48-specific polyubiquitin chains from ENO1. This effect was abolished when the K48R mutant plasmid was introduced (Fig. 5D). These results demonstrate that UCHL3 specifically targets and removes K48-mediated polyubiquitination of ENO1.To further explore the deubiquitination sites of ENO1 targeted by UCHL3, we performed immunoprecipitation to collect ENO1 protein, followed by SDS-PAGE separation. The gel region corresponding to ENO1 was subjected to mass spectrometry, which identified lysine residues at positions 89 and 92 as ubiquitination sites (Fig. 5E). In vivo ubiquitination assays confirmed that UCHL3 primarily removes polyubiquitin chains from lysine 92 on ENO1 (Fig. 5F). To validate these findings, we co-transfected GC cells with UCHL3 overexpression plasmids together with either wild-type ENO1 or its mutants (K92R or K89R). Our results revealed that UCHL3 overexpression stabilized wild-type ENO1 but failed to stabilize the K92R mutant, which exhibited significantly reduced half-life. Importantly, the stability of the K89R mutant was comparable to that of wild-type ENO1 (Supplementary Fig. S3E, F). These findings confirm that UCHL3 specifically removes polyubiquitin chains from ENO1 primarily at lysine 92.

### ENO1 is a key mediator of UCHL3-regulated AKT/CCND1 signaling to promote GC progression and shows a positive correlation with UCHL3 expression

**Fig. 6:**
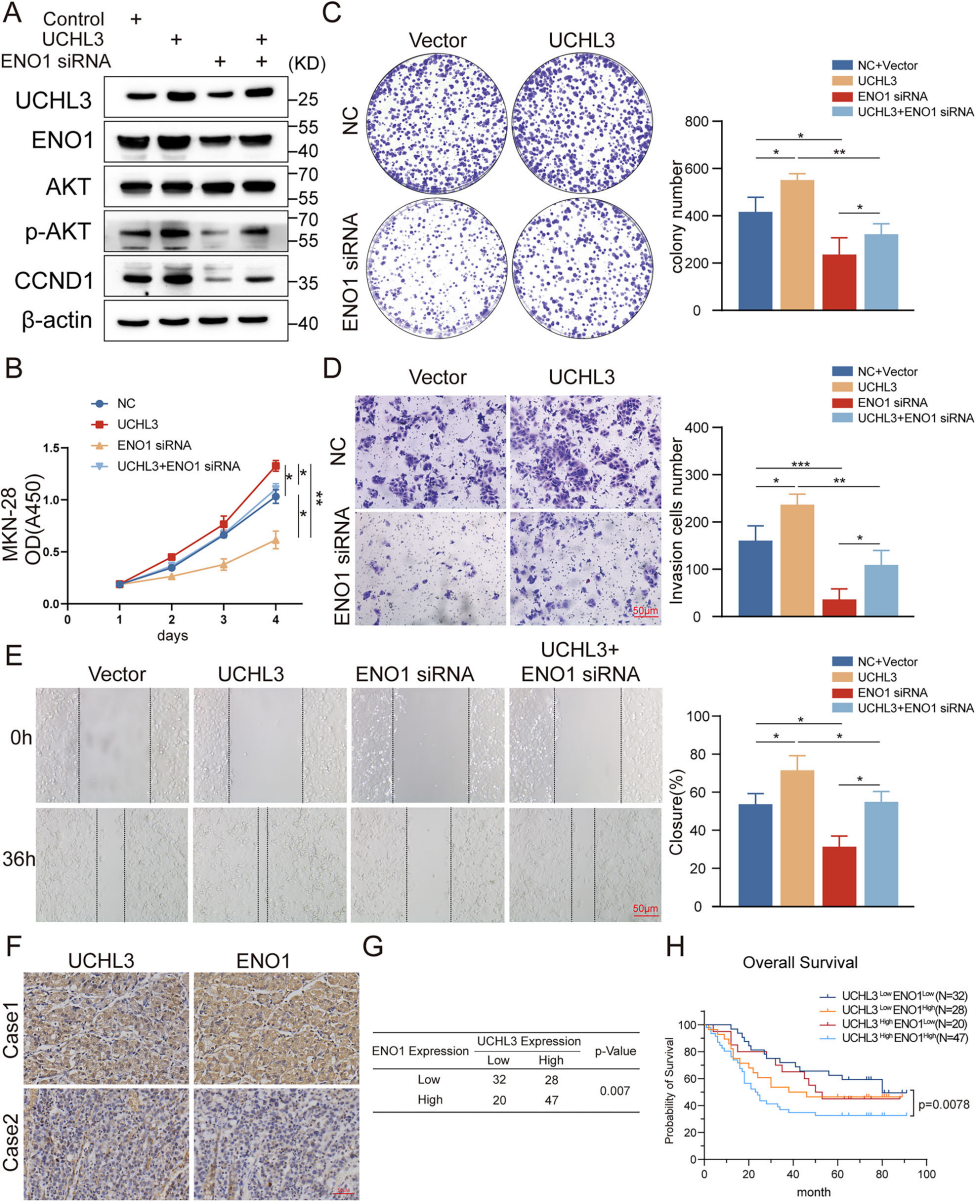
ENO1 is a key mediator of UCHL3-regulated AKT/CCND1 signaling to promote GC progression and shows positive correlation with UCHL3 expression. **A** Western blot analysis of ENO1, AKT, p-AKT(s473), and CCND1 expression in MKN-28 cells transfected with UCHL3 overexpression plasmid and/or ENO1 siRNA as indicated. **B**–**E** Assessment of GC cell proliferation, invasion, and migration following transfection with UCHL3 overexpression plasmid and/or ENO1 siRNA using CCK-8 assay (**B**), colony formation assay (**C**), Transwell invasion assay (**D**), and wound healing assay (**E**). All experiments were repeated at least three times. **F** Representative IHC images of UCHL3 and ENO1 in two cases of GC patients. **G** Correlation between UCHL3 and ENO1 expression in GC tissues was analyzed by chi-square test. **H** Survival analysis of the 127 GC patients based on IHC scores of UCHL3 and ENO1. One-way ANOVA: *P < 0.05, **P < 0.01, ***P < 0.001.

To explore the role of ENO1 in UCHL3-mediated GC progression, we co-transfected UCHL3 overexpression plasmids and ENO1 siRNA into MKN-28 GC cells and observed changes in the AKT/CCND1 signaling axis and cellular functions. As shown in Fig. 6A, UCHL3 overexpression activated the AKT/CCND1 signaling axis, while ENO1 siRNA partially reversed UCHL3’s activation of the AKT/CCND1 signaling axis. Results from CCK-8 assays, colony formation assays, transwell invasion assays, and wound healing assays also indicated that ENO1 siRNA significantly inhibited UCHL3’s promotion of GC cell proliferation, invasion, and migration abilities (Fig. 6B–E). Furthermore, rescue experiments in UCHL3-knockout cells revealed that ENO1 overexpression not only restored cellular phenotypes but also rescued the impaired AKT/CCND1 signaling pathway (Supplementary Fig. S4). To clinically validate the UCHL3-ENO1 relationship, we performed ENO1 immunohistochemistry in our cohort of 127 GC tissues. The results demonstrated a significant positive correlation between UCHL3 and ENO1 expression (*P* = 0.007), with patients exhibiting concurrent high expression of both markers showing significantly worse prognosis compared to those with low expression of both proteins (Fig. 6F–H). Collectively, these findings establish ENO1 as a crucial mediator of UCHL3’s oncogenic effects through AKT/CCND1 signaling activation in GC progression, with their coordinated expression patterns carrying clinical prognostic significance.

### UCHL3 promotes tumor progression in vivo

**Fig. 7:**
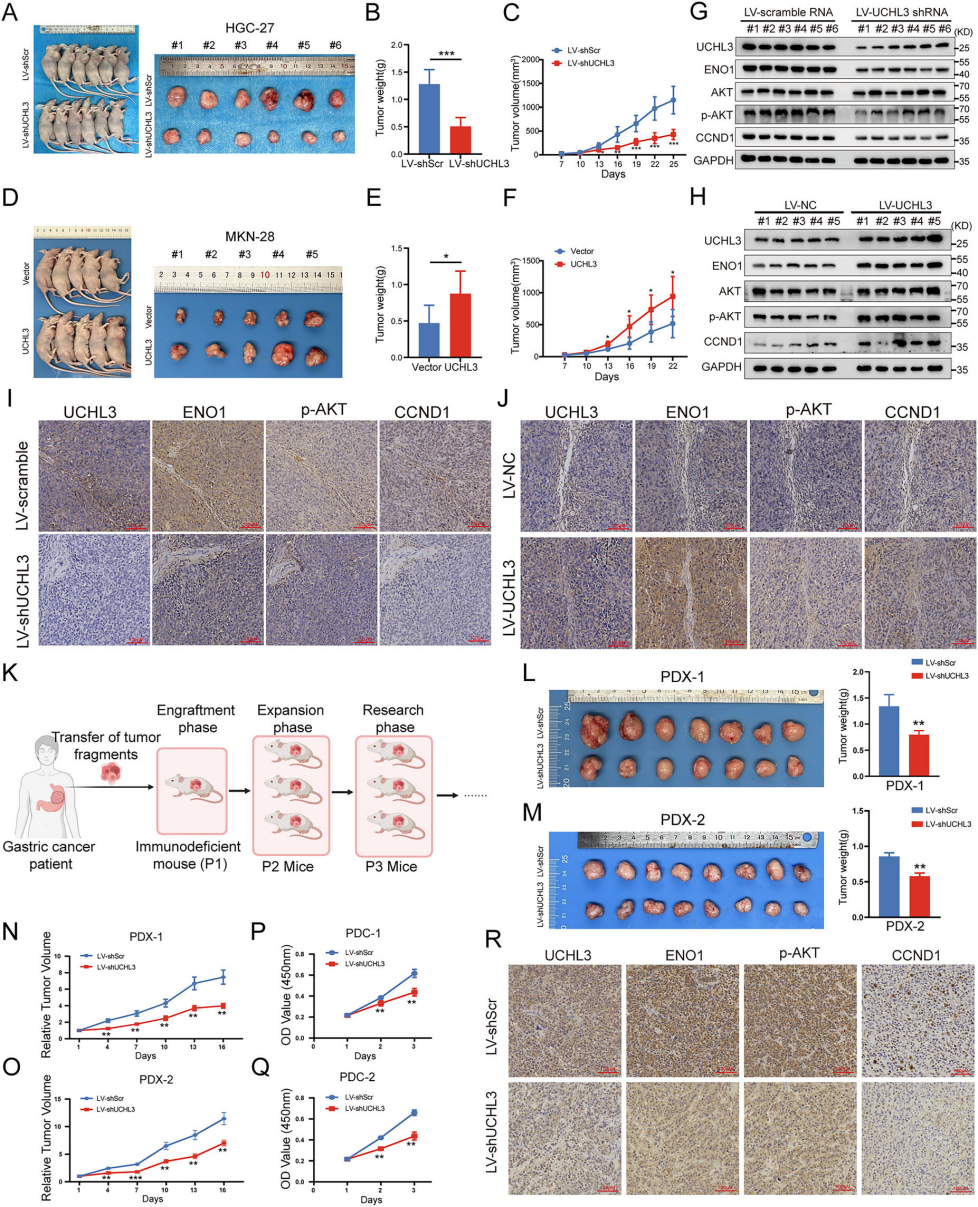
UCHL3 promotes tumor progression in vivo. **A**–**C** HGC-27 cells with stable knockdown of UCHL3 were constructed using lentiviral vectors, alongside a negative control group. Cells were injected into the flanks of Balb/c nude mice to observe in vivo tumor growth. Tumor volumes were measured (**C**), and on day 25, the mice were euthanized, tumors were excised for photography (**A**), and weighed (**B**). **D**–**F** MKN-28 cells with stable overexpression of UCHL3 were also created using lentiviral vectors, following the same experimental procedure. Tumor volumes were measured (**F**), and on day 22, the tumors were excised for photography (**D**) and weighed (**E**). **G**–**J** Immunohistochemistry and Western blotting were conducted on xenograft tumor tissues to assess the impact of UCHL3 silencing or UCHL3 overexpression on the expression of ENO1, p-AKT, and CCND1 proteins. **K** Tumor tissues from two GC patients were implanted subcutaneously into mice to establish PDX models. **L**–**R** Injections of shScramble and shUCHL3 lentivirus were administered intra-tumorally in PDX-1 and PDX-2, respectively. Tumors were excised, photographed, and weighed at the endpoint of the experiment (**L**, **M**). Tumor growth curves of PDX-1 and PDX-2 mice (**N**, **O**). CCK-8 assay to evaluate the proliferation ability of primary GC cells derived from PDX-1 (PDC-1) and PDX-2 (PDC-2) following infection with shScramble and shUCHL3 lentivirus (**P**, **Q**). Immunohistochemistry was conducted on PDX xenograft tumor tissues to evaluate the effects of UCHL3 knockdown on the expression levels of ENO1, p-AKT, and CCND1 proteins. Student’s *t* test: *P < 0.05, **P < 0.01, ***P < 0.001.

To further validate our findings, we established cell line-derived xenograft (CDX) and PDX models in Balb/C nude mice by injecting tumor cells or patient-derived GC tissues subcutaneously. In xenografts constructed with HGC-27 cells, silencing UCHL3 resulted in reduced proliferation, along with smaller tumor size and weight (Fig. 7A–C). Conversely, increasing UCHL3 expression in MKN-28 cells promoted in vivo proliferation of GC cells (Fig. 7D–F). Immunohistochemistry and western blotting analysis of CDX model tumor tissues revealed reduced levels of ENO1, p-AKT, and CCND1 in UCHL3-silenced tumors, while these levels were elevated in tumors overexpressing UCHL3 (Fig. 7G– J). These experiments confirmed that UCHL3 promotes GC proliferation and activates the ENO1/AKT/CCND1 signaling axis in vivo. In addition, we constructed two PDX models as shown in Fig. 7K and observed that treatment with shUCHL3 lentivirus significantly slowed tumor growth, resulting in smaller tumor size and weight compared to negative controls (Fig. 7L–O). Furthermore, infection with shUCHL3 lentivirus reduced the proliferation of primary patient-derived tumor cells (PDC) (Fig. 7P, Q). Consistent with the results from UCHL3-silenced CDX models, IHC analysis of the PDX models revealed decreased UCHL3 expression, accompanied by reduced levels of ENO1, p-AKT, and CCND1 (Fig. 7R). These findings underscore the potential clinical relevance of UCHL3-targeted therapy.

### TCID suppresses UCHL3 function and increases GC cell sensitivity to CDK4/6 inhibitors

**Fig. 8:**
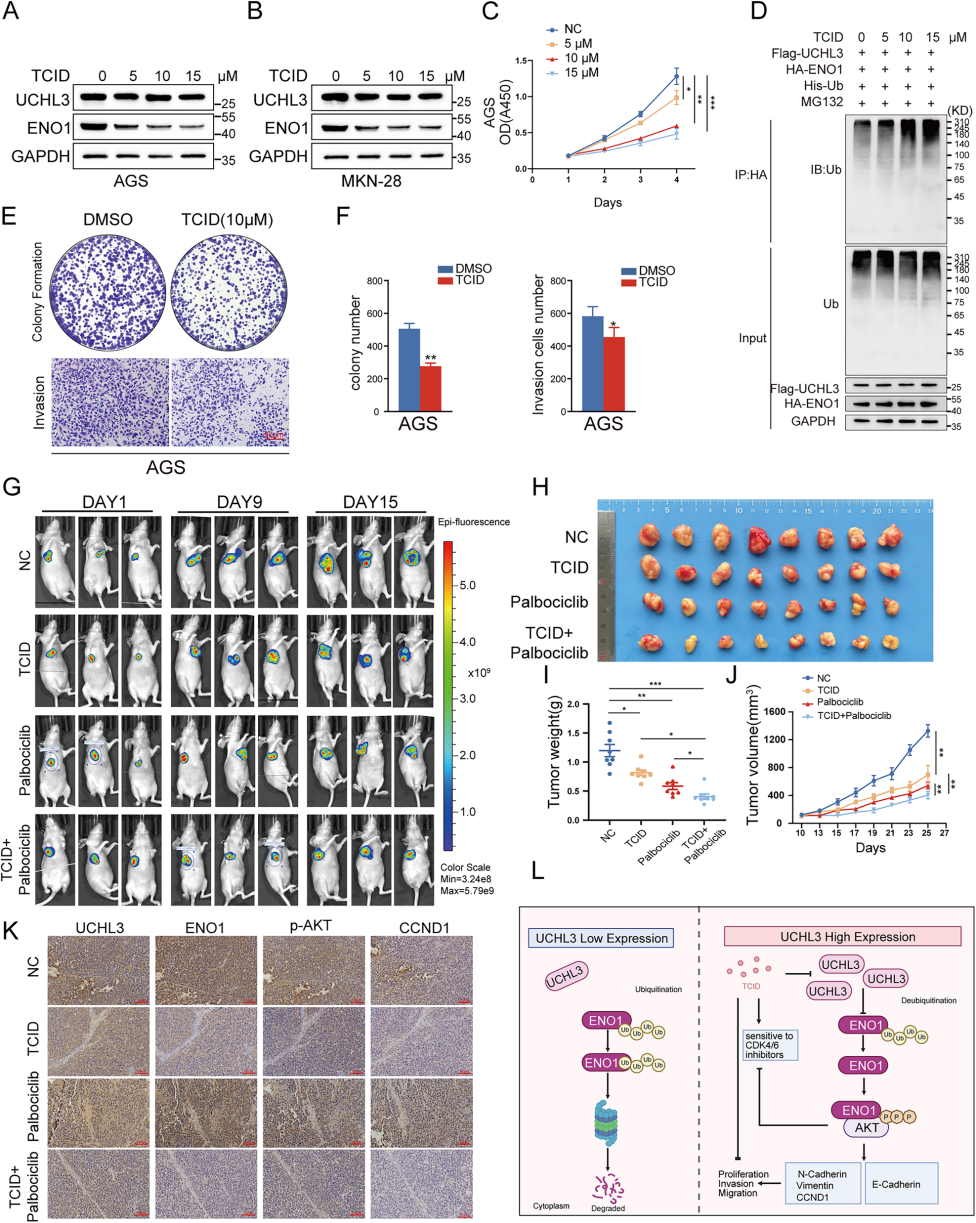
TCID suppresses UCHL3 function, and increases GC cell sensitivity to CDK4/6 inhibitors. **A**, **B** Western blotting analysis of UCHL3 and ENO1 protein expression in AGS and MKN-28 cells treated with 0, 5, 10, and 15 μM TCID (a UCHL3 inhibitor). **C** AGS cells were treated with 0, 5, 10, and 15 μM TCID, and cell viability was assessed using the CCK-8 assay. **D** 293T cells were transfected with Flag-UCHL3, His-Ub, and HA-ENO1, followed by treatment with 0, 5, 10, and 15 μM TCID, and Western blotting was performed to detect the ubiquitination level of ENO1. **E**, **F** AGS cells were treated with 0 and 10 μM TCID, and changes in proliferation and invasion abilities of GC cells were assessed using colony formation (**E**) and Transwell invasion assays (**F**). **G**–**K** HGC-27 cells were infected with a GFP-labeled empty plasmid using lentiviral vectors, expanded, and implanted subcutaneously into the right flanks of nude mice. When the tumors reached 100–150 mm³, the mice were treated with the following regimens: placebo (intraperitoneal saline every two days and oral Lactated Ringer’s solution daily), TCID monotherapy (10 mg/kg, dissolved in DMSO and further diluted with saline, administered intraperitoneally every two days, and oral Lactated Ringer’s solution daily), Palbociclib monotherapy (150 mg/kg, prepared in Lactated Ringer’s solution, administered orally once daily), or combination therapy (TCID, 10 mg/kg intraperitoneally every two days plus Palbociclib, 150 mg/kg orally daily). In vivo imaging was performed every two days post-treatment. The figure shows representative fluorescence images from in vivo imaging of a subset of mice on days 1, 9, and 15 post-treatment (**G**). Tumors were excised, photographed (**H**), and weighed (**I**) at the end of the experiment. Tumor volumes were measured every two days post-treatment (**J**). **K** Immunohistochemistry was used to detect the expression of UCHL3, p-AKT, ENO1, and CCND1 in tumor tissues from each group. **L** Schematic Diagram: UCHL3 overexpression stabilizes ENO1 by deubiquitination, leading to enhanced AKT phosphorylation and activation of downstream target gene CCND1, promoting proliferation, invasion, and migration of GC cells. The small-molecule inhibitor TCID suppresses UCHL3 function and sensitizes GC cells to CDK4/6 inhibitors.

4,5,6,7-Tetrachloroindan-1,3-dione (TCID) is a potent and selective inhibitor of UCHL3 [31, 32]. However, its effect on UCHL3-mediated deubiquitination of ENO1 and its impact on GC cell proliferation and invasion remain unclear. Based on a literature review and manufacturer recommendations, we designed a TCID treatment regimen for AGS GC cells at concentrations of 0, 5, 10, and 15 μM [28]. While TCID did not affect UCHL3 protein expression, it significantly reduced ENO1 protein levels in a dose-dependent manner (Fig. 8A, B) and inhibited UCHL3-mediated deubiquitination of ENO1 (Fig. 8D). In addition, TCID suppressed the proliferation and invasion of AGS cells (Fig. 8C, E, F). These results suggest that TCID effectively targets UCHL3 function, thereby inhibiting GC cell growth and invasion.

CDK4/6 inhibitors, such as palbociclib, are promising anti-tumor agents, but easily resulted in resistance due to the overexpression of CCND1 and CCNE [33, 34]. Combining therapies is an effective strategy to overcome this resistance [33, 35]. Our study suggests that UCHL3 promotes CCND1 expression, potentially contributing to CDK4/6 inhibitor resistance. To explore this, we investigated whether inhibiting UCHL3 with the small-molecule inhibitor TCID could enhance the sensitivity of GC cells to the CDK4/6 inhibitor palbociclib both in vitro and in vivo. Functional assays showed that TCID, either alone or in combination with palbociclib, significantly inhibited cell proliferation, with the combination treatment demonstrating the highest efficacy. While palbociclib alone had minimal effects on cell invasion, the combination with TCID was more effective (Supplementary Fig. 4A–C). In a mouse model, the combination of TCID and palbociclib inhibited tumor growth more effectively than either drug alone (Fig. 8G–J). IHC analysis of tumor tissues from the mouse model revealed that UCHL3 expression in the TCID group remained unchanged. However, its downstream targets, ENO1, p-AKT, and CCND1, were significantly reduced. Interestingly, although tumor size decreased in the palbociclib group, CCND1 expression was slightly higher than in the control group (Fig. 8K), further supporting the notion that increased CCND1 expression may contribute to palbociclib resistance. T These findings suggest that TCID enhances the anti-tumor efficacy of palbociclib in GC.

## Discussion

Advanced GC typically carries a bleak prognosis. However, targeted therapies like HER2 and VEGFR inhibitors, along with immunotherapies such as PD-1/PD-L1 inhibitors, have shown significant clinical advancements, offering new hope for these patients [36]. Further exploring GC characteristics and identifying treatment targets are vital for improving patient outcomes. Through bioinformatics analysis and tissue samples, our study found that UCHL3 is amplified and overexpressed in GC, correlating with aggressive behaviors and poor prognosis. This aligns with Peng et al.‘s findings of elevated UCHL3 expression in GC tissues and its association with lymph node and distant metastasis in 47 pairs of tissue samples [37]. Our study, with a larger sample size, reinforces these conclusions.

Our study revealed that UCHL3 enhances proliferation, invasion, and migration of GC cells, accelerating the cell cycle and inducing EMT. Similar effects of UCHL3 on malignancy have been observed in other cancers. For instance, UCHL3 deficiency sensitizes thyroid cancer cells to chemotherapy [28], while its overexpression induces treatment resistance in breast cancers [38]. This suggests UCHL3 as a potential therapeutic target for GC. PDX models, crucial in cancer research, were utilized in our study. By downregulating UCHL3 expression in GC PDX models, we observed slower tumor growth, further confirming UCHL3’s therapeutic potential. However, further translational research and clinical validation are needed to ascertain its impact on patient survival.

We identified the binding association between UCHL3 and ENO1 in GC through techniques such as mass spectrometry, Co-IP, and GST-pull down experiments. Previous studies have indicated that ENO1 serves as an activator of the PI3K/AKT signaling pathway [22–24]. In addition, Deng et al. showed ENO1 binds to AKT, activating CCND1 expression downstream, while CCDC65 assists ENO1 degradation via FBXW7, thereby inhibiting GC progression [25]. Sun et al. found ENO1 overexpression boosts AKT phosphorylation, driving GC growth [24]. Based on these, we speculate that UCHL3 activates the AKT/CCND1 pathway via ENO1, and subsequent rescue experiments confirm ENO1’s pivotal role in UCHL3-mediated AKT/CCND1 signaling regulation for GC progression. However, ENO1 siRNA fails to fully reverse the downstream protein and phenotypic changes induced by UCHL3 overexpression. Immunofluorescence experiments reveal ENO1’s presence solely in the cytoplasm of GC cells, while UCHL3 is detected in both the cytoplasm and nucleus, suggesting UCHL3 may exert its effects through other pathways. The studies by Liu et al. and Ryotaro Nishi et al. demonstrated that UCHL3’s involvement in DNA homologous recombination (HR) and non-homologous end joining (NHEJ) processes, suggesting that UCHL3 interacts with relevant molecules in the nucleus, leading to alterations in DNA repair functionality [38, 39]. This finding aligns with our observation of UCHL3’s localization in both the cytoplasm and nucleus in Fig. 4G. In addition, Peng et al. uncovered UCHL3’s non-deubiquitination role in activating IGF2 transcription, promoting GC metastasis [37]. Our study further clarifies UCHL3’s multifaceted contribution to tumor progression, providing novel insights into its regulatory network. Moreover, we identified the binding region and functional sites of UCHL3 with ENO1, laying the groundwork for future drug development endeavors.

Consistent with prior research, our experiments confirmed that TCID inhibits UCHL3, leading to ENO1 ubiquitination and suppressing GC cell proliferation and invasion. CDK4/6 inhibitors are mainly used for breast cancer, but they show promise in other cancers too, such as non-small-cell lung cancer patients and melanoma patients [11, 40]. In prior studies, CDK4/6 inhibitors resulted in disease control rates (DCR) of 49% (*n* = 68) and 44% (*n* = 18) in non-small cell lung cancer and melanoma patients, respectively [41, 42]. The objective response rate (ORR) in head and neck squamous cell carcinoma patients was 39% (*n* = 62) [43]. Our experiments showed that palbociclib effectively reduced the proliferation of GC cells in vivo and in vitro, indicating its therapeutic potential in GC. Nonetheless, various factors contribute to CDK4/6 resistance, including loss of Rb gene, overexpression of cyclin E, cyclin D1, CDK4/6, and activation of bypass pathways like FGFR and PI3K/Akt/mTOR signaling [40, 44, 45], our IHC staining results from the palbociclib-treated mouse group also indirectly suggest that the acquired increase in CCND1 expression might be one of the reasons for resistance. Combination therapy is being investigated to counter resistance. For instance, PI3K inhibitors decrease Cyclin D1 expression, restoring sensitivity of breast cancer cells to CDK4/6 inhibitors [46]. mTOR1/2 inhibitors reduce levels of cyclin D1, phosphorylated Rb, and E2F expression, effectively reversing resistance to CDK4/6 inhibitors [47]. Professor Shen has conducted a preliminary exploration of the role of CDK4/6 inhibitors in GC, finding that the disease control rate of CDK4/6 inhibitors in combination with Pyrotinib in GC is as high as 93.8% [13]. In our study, we found that the therapeutic effect of palbociclib alone was inferior to the combination of palbociclib and TCID, indicating that TCID enhances the sensitivity of GC cells to palbociclib, further supporting the combination therapy strategy of CDK4/6 inhibitors.

In summary, our study elucidates the oncogenic role of UCHL3 in GC through clinical samples and in vivo and in vitro models. Mechanistically, UCHL3 removes K48-linked polyubiquitin chains from ENO1, increasing ENO1 protein expression and activating the AKT/CCND1 pathway, thus promoting GC progression. Furthermore, inhibition of UCHL3 by TCID enhances the sensitivity of GC cells to CDK4/6 inhibitors. These findings highlight UCHL3 as a potential therapeutic target for GC (Fig. 8L).

## Supplementary information

Supplementary material

Supplemental Material-Raw data

## Data Availability

The authors declare that the data support the findings of this study are available from the authors upon reasonable request. The full-length, uncropped Western blot images are available in the “Supplemental Material-Raw data”.
