# Supplementary material for: UCHL3 depletion inhibits gastric cancer progression and enhances palbociclib sensitivity by regulating the AKT/CCND1 signaling axis via ENO1 ubiquitination
Source: Cell Death Dis. 2025 Nov 21;16(1):850. doi: 10.1038/s41419-025-08153-3 (PMC12638999; doi:10.1038/s41419-025-08153-3)
Supplement: Supplementary file 1 — Supplementary material [file 41419_2025_8153_MOESM1_ESM.docx]

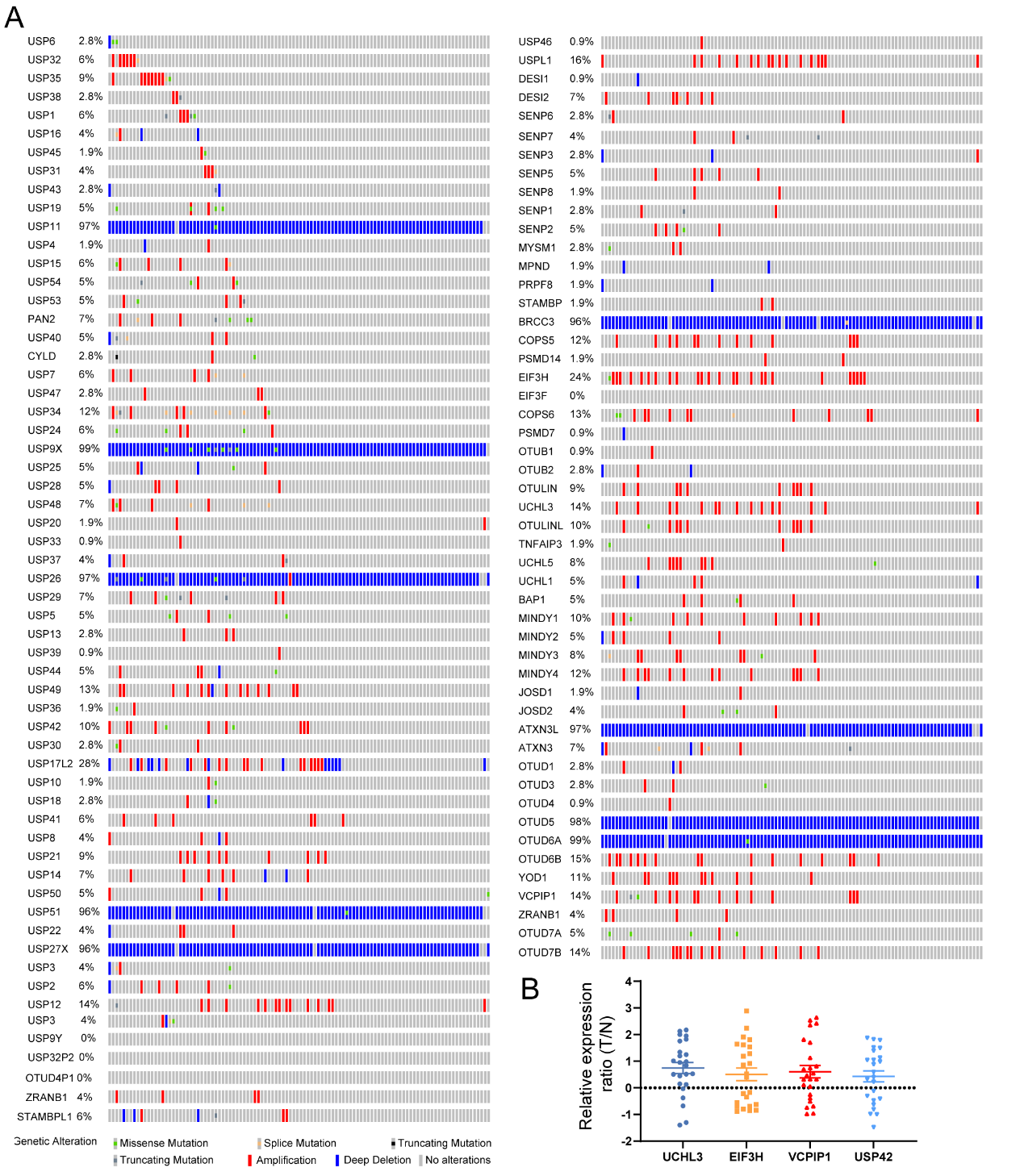
**Supplementary Materials**

**Fig. S1 Gene mutations** **status of deubiquitinating enzymes in gastric cancer (GC). A** Exploration of gene mutations in all 110 deubiquitinating enzymes in GC using the cBioPortal website (https://www.cbioportal.org/, dataset: OncoSG, 2018). **B** Expression ratios of 4 candidate genes between tumor and adjacent normal tissues in 24 paired GC specimens.


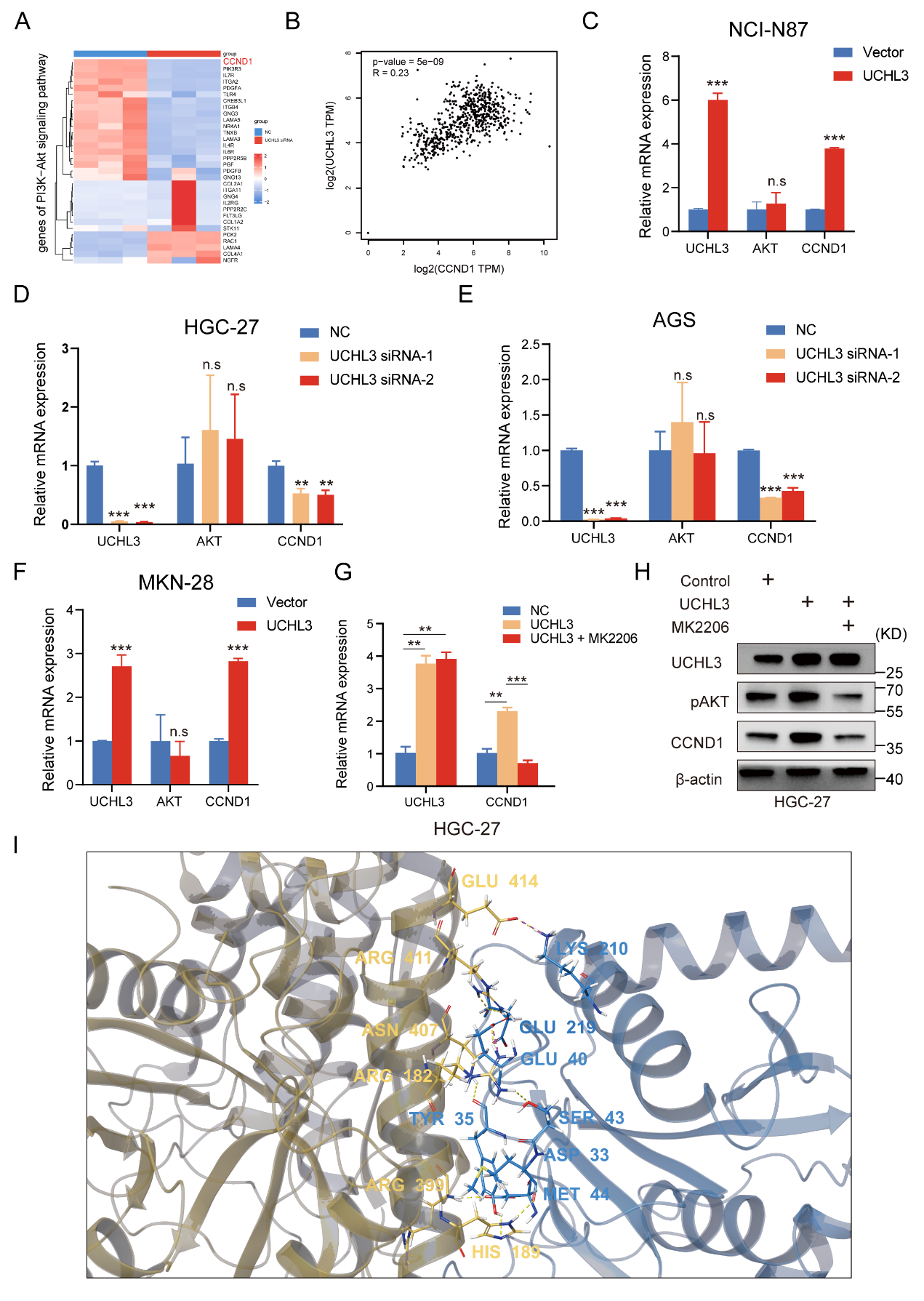


**Fig. S2 UCHL3** **regulates the AKT/CCND1 signaling pathway.**

**A** Heatmap showing differentially expressed genes enriched in the AKT signaling pathway. **B** The correlation between UCHL3 and CCND1 expression detected in TCGA GC data. **C, D** RT-qPCR analysis of the impact of UCHL3 upregulation on the AKT/CCND1 signaling axis in HGC-27 and AGS cells. **E, F** RT-qPCR analysis of the impact of UCHL3 silencing on the AKT/CCND1 signaling axis in MKN-28 cells. **G, H** Effect of AKT phosphorylation inhibitor MK-2206 on UCHL3-mediated CCND1 regulation assessed by RT-qPCR (G) and Western blotting (H). **I** The molecular docking simulation (enlarged view) of UCHL3-ENO1 interaction identifies the specific chemical bonding patterns at their interface. Student’s t test: *p<0.05, **p<0.01, ***p<0.001.


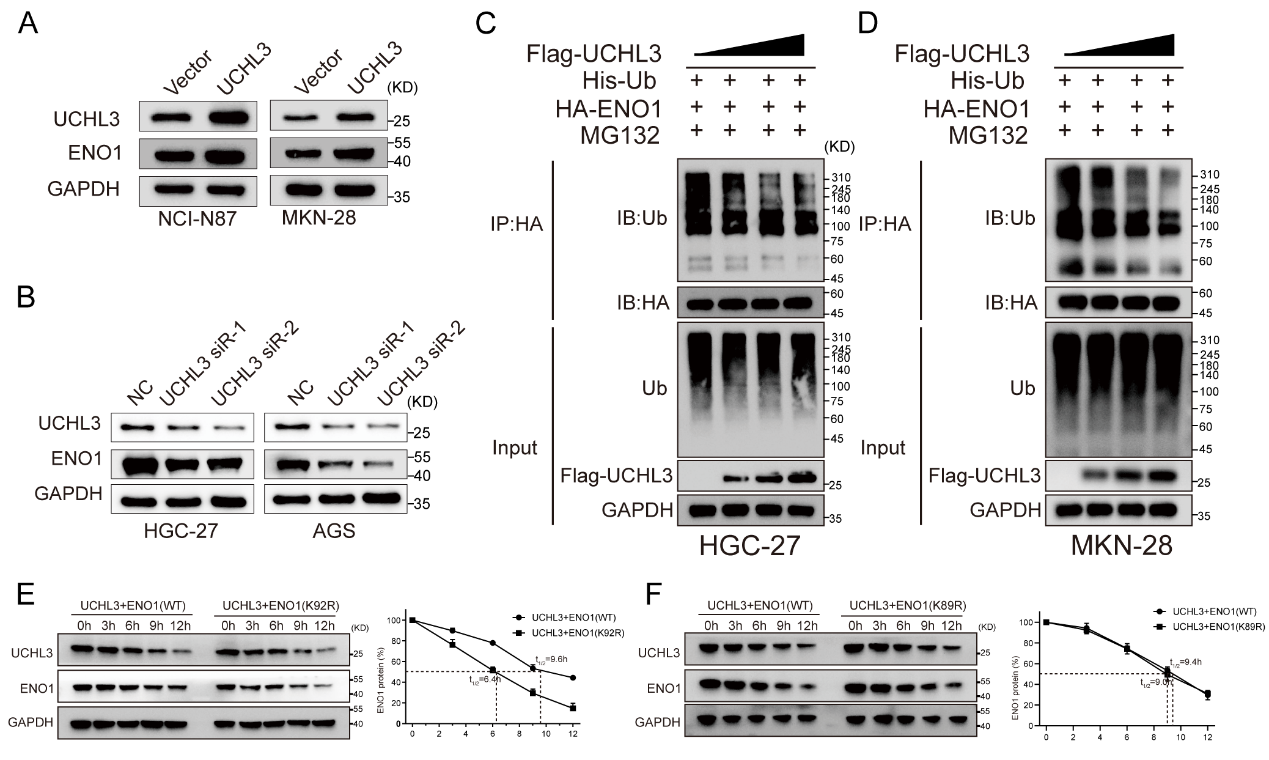


**Fig. S****3 Mass spectrometry (MS) identifies potential proteins binding to UCHL3.**

**A, B** Western blot analysis of ENO1 protein expression in NCI-N87, MKN-28, HGC-27, and AGS cells with either silenced or overexpressed UCHL3. **C, D** HGC-27 and MKN-28 were transfected with different doses of UCHL3 overexpression plasmids, and in vivo ubiquitination assays were performed to assess the effect of UCHL3 expression levels on the ubiquitination of ENO1. **E, F** Protein stability assays in gastric cancer cells co-transfected with UCHL3 overexpression plasmids plus either wild-type ENO1 or its mutants (K92R/K89R), demonstrating comparable stability between K89R mutant(E) or K92R mutant(F) and wild-type ENO1.


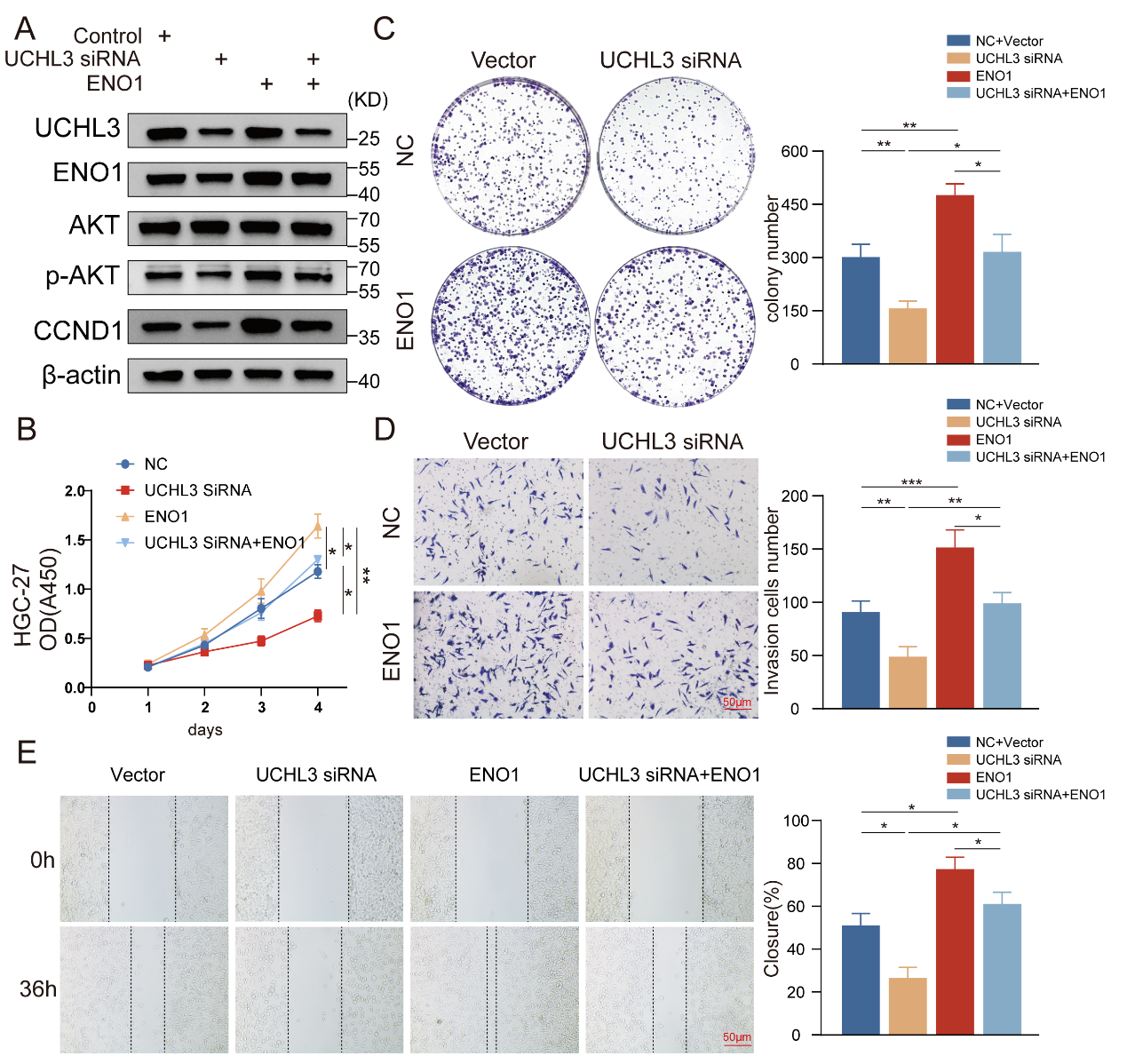


**Fig. S4 ENO1 is a key mediator of UCHL3-regulated AKT/CCND1 signaling to promote GC progression**

**A** Western blot analysis of ENO1, AKT, p-AKT(s473), and CCND1 expression in MKN-28 cells transfected with UCHL3 siRNA and/or ENO1 overexpression plasmid as indicated. **B-E** Assessment of GC cell proliferation, invasion, and migration following transfection with UCHL3 siRNA and/or ENO1 overexpression plasmid using CCK-8 assay (**B**), colony formation assay (**C**), Transwell invasion assay (**D**), and wound healing assay (**E**).All experiments were repeated at least three times.One-way ANOVA: *p<0.05, **p<0.01, ***p<0.001.


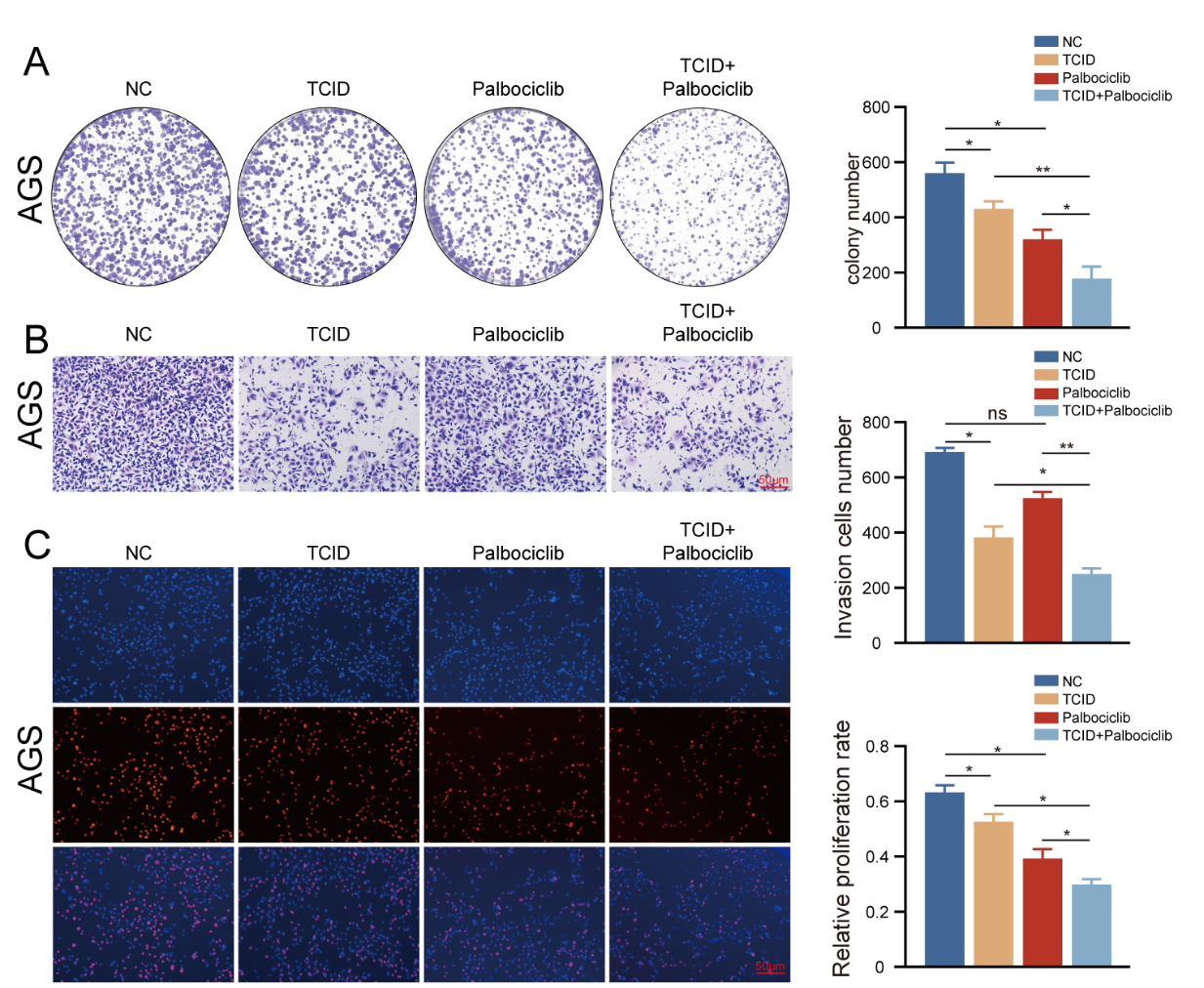


**Fig. S5 TCID increases GC cell sensitivity to CDK4/6 inhibitors.**

**A-C** Colony formation, Transwell invasion assays and EdU assay were used to validate the effects of TCID monotherapy, Palbociclib monotherapy, and combination therapy on the proliferation and invasion abilities of GC cells. to validate the effects of TCID monotherapy, palbociclib monotherapy, and combination therapy on the proliferation of GC cells.

**Supplementary Table S1: The sequences of UCHL3 siRNAs and ENO1 siRNAs used in this study.**

| **Gene** | **Sequences for siRNA** |
| --- | --- |
| UCHL3 siRNA-1 | Sense: 5′-GGCGGAAGCCAUUUCCAAUTT-3′ |
|  | Antisense: 5′-AUUGGAAAUGGCUUCCGCCTT-3′ |
| UCHL3 siRNA-2 | Sense: 5′-CCCUGAUGAACUAAGAUUUTT-3′ |
|  | Antisense: 5′-AAAUCUUAGUUCAUCAGGGTT-3′ |
| ENO1 siRNA | Sense:5’ -GCAUUGGAGCAGAGGUUUATT-3’ |
|  | Antisense: 5′- UAAACCUCUGCUCCAAUGCTT-3′ |

**Supplementary Table S2: The specific information for the primary antibodies is as follows:**

| **Primary antibodies** | **Details** |
| --- | --- |
| UCHL3 | 1:1000, 12384-1-AP, proteintech, Wuhan, China |
| UCHL3 | 1:200, sc-100340, Santa Cruz Biotech., CA, USA |
| ENO1 | 1:10000, 11204-1-AP, proteintech, Wuhan, China |
| ENO1 | 1:200, sc-100812, Santa Cruz Biotech., CA, USA |
| Phospho-AKT (Ser473) | 1:1000, 80455-1-RR, proteintech, Wuhan, China |
| AKT | 1:4000, 10176-2-AP, proteintech, Wuhan, China |
| Cyclin D1 | 1:5000, ab134175, Abcam, Cambridge, UK |
| GAPDH | 1:15000, 60004-1-Ig, proteintech, Wuhan, China |
| β-actin | Zhongshan Golden Bridge, Beijing, China |
| ubiquitin | 1:1000, 10201-2-AP, proteintech, Wuhan, China |
| GFP tag | 1:10000, 66002-1-Ig, proteintech, Wuhan, China |
| HA tag | 1:5000, M20021, abmart, Shanghai, China |
| HA tag | 1:1000, #561,MBL, Nagoya, Japan |
| Flag tag | 1:1000, ab205606, Abcam, Cambridge, UK |

| **Gene** | **Primer sequences (5'-3')** |
| --- | --- |
| UCHL3 | Forward: GGAAGAGTCCAAGCGTGAGG |
|  | Reverse: GAAACTGGTTGGTGACCCTCT |
| ENO1 | Forward: GCCGTGAACGAGAAGTCCTG |
|  | Reverse: ACGCCTGAAGAGACTCGGT |
| AKT | Forward: GTCAAAGAAGTCAAAGGGGCTG |
|  | Reverse: TCTTGATGTACTCCCCTCGTT |
| CCND1 | Forward: AAGGAGCTGGGATTCGATG |
|  | Reverse: AGGCTCCATTCCAAAAACAG |
| GAPDH | Forward: 5’-CACCCACTCCTCCACCTTTG-3’ |
|  | Reverse: 5’-CCACCACCCTGTTGCTGTAG-3’ |

**Supplementary Table S3. Primer sequences used in qRT-PCR assays.**

**Supplementary Table 4:** **Proteomic analysis performed by LC–MS/MS**

| Accession | Gene names | MW [kDa] | Protein score | Sequence coverage (%) | Unique Peptides | Peptides | PSMs | Abundances |
| --- | --- | --- | --- | --- | --- | --- | --- | --- |
| P14618 | PKM | 57.9 | 1377 | 60 | 24 | 24 | 31 | 6.95e+08 |
| P15374 | UCHL3 | 26.2 | 1191 | 80 | 15 | 15 | 29 | 2.26e+09 |
| P35579 | MYH9 | 226.4 | 1107 | 18 | 24 | 24 | 26 | 1.99e+08 |
| P07437 | TUBB | 49.6 | 975 | 59 | 7 | 18 | 26 | 7.92e+08 |
| P06733 | ENO1 | 47.1 | 836 | 36 | 12 | 13 | 19 | 5.55e+08 |
| P07900 | HSP90AA1 | 84.6 | 829 | 27 | 8 | 16 | 19 | 2.00e+08 |
| P08238 | HSP90AB1 | 83.2 | 767 | 31 | 9 | 19 | 20 | 5.61e+08 |
| P02768 | ALB | 69.3 | 585 | 32 | 16 | 16 | 18 | 4.11e+08 |
| P11021 | HSPA5 | 72.3 | 474 | 24 | 9 | 11 | 12 | 7.96e+07 |
| P68104 | EEF1A1 | 50.1 | 460 | 29 | 8 | 8 | 12 | 1.02e+09 |
| P13639 | EEF2 | 95.3 | 444 | 19 | 11 | 11 | 13 | 1.26e+08 |
| P04075 | ALDOA | 39.4 | 367 | 30 | 7 | 7 | 9 | 1.37e+08 |
| P24844 | MYL9 | 19.8 | 365 | 45 | 1 | 5 | 8 | 9.12e+06 |
| P38646 | HSPA9 | 73.6 | 357 | 16 | 8 | 8 | 8 | 7.71e+07 |
| P61978 | HNRNPK | 50.9 | 334 | 23 | 7 | 7 | 8 | 6.44e+07 |
| P06748 | NPM1 | 32.6 | 315 | 12 | 2 | 2 | 4 | 3.85e+07 |
| P01614 | IGKV2D-40 | 13.3 | 300 | 11 | 1 | 1 | 7 | 3.54e+09 |
| P25705 | ATP5F1A | 59.7 | 269 | 16 | 6 | 6 | 7 | 1.61e+08 |
| P30086 | PEBP1 | 21 | 258 | 36 | 4 | 4 | 4 | 2.07e+07 |
| P08865 | RPSA | 32.8 | 254 | 11 | 2 | 2 | 3 | 1.73e+07 |
| P13929 | ENO3 | 47 | 239 | 8 | 1 | 2 | 3 | 6.09e+07 |
| P06576 | ATP5F1B | 56.5 | 233 | 11 | 4 | 4 | 5 | 5.89e+07 |
| P30101 | PDIA3 | 56.7 | 215 | 13 | 6 | 6 | 6 | 5.30e+07 |
| P62258 | YWHAE | 29.2 | 213 | 24 | 4 | 6 | 6 | 1.51e+08 |
| P50990 | CCT8 | 59.6 | 211 | 10 | 4 | 4 | 5 | 3.89e+07 |
| P23528 | CFL1 | 18.5 | 206 | 32 | 4 | 4 | 5 | 6.20e+07 |
| P29401 | TKT | 67.8 | 195 | 13 | 5 | 5 | 5 | 3.47e+07 |
| Q13310 | PABPC4 | 70.7 | 191 | 9 | 1 | 4 | 4 | 6.13e+06 |
| P37802 | TAGLN2 | 22.4 | 177 | 28 | 4 | 4 | 4 | 3.91e+07 |
| P62829 | RPL23 | 14.9 | 170 | 13 | 2 | 2 | 4 | 5.52e+07 |
| P27348 | YWHAQ | 27.7 | 167 | 16 | 2 | 4 | 4 | 8.35e+06 |
| P02786 | TFRC | 84.8 | 164 | 8 | 5 | 5 | 5 | 3.32e+07 |
| P40926 | MDH2 | 35.5 | 151 | 15 | 3 | 3 | 3 | 2.29e+07 |
| Q13200 | PSMD2 | 100.1 | 150 | 4 | 3 | 3 | 4 | 1.01e+07 |
| P62987 | UBA52 | 14.7 | 130 | 23 | 2 | 2 | 3 | 3.57e+07 |
| P49368 | CCT3 | 60.5 | 125 | 6 | 3 | 3 | 3 | 3.90e+07 |
| Q92841 | DDX17 | 80.2 | 123 | 5 | 2 | 3 | 3 | 1.31e+07 |
| P26373 | RPL13 | 24.2 | 121 | 18 | 4 | 4 | 4 | 1.01e+08 |
| P63173 | RPL38 | 8.2 | 120 | 33 | 2 | 2 | 3 | 3.94e+07 |
| Q07021 | C1QBP | 31.3 | 119 | 10 | 2 | 2 | 3 | 1.80e+07 |
| P62851 | RPS25 | 13.7 | 118 | 17 | 3 | 3 | 3 | 9.27e+07 |
| P62273 | RPS29 | 6.7 | 110 | 32 | 2 | 2 | 3 | 4.43e+08 |
| P09429 | HMGB1 | 24.9 | 109 | 19 | 3 | 3 | 3 | 2.54e+07 |
| P13010 | XRCC5 | 82.7 | 108 | 5 | 3 | 3 | 3 | 1.23e+07 |
| P16402 | H1-3 | 22.3 | 106 | 11 | 2 | 2 | 2 | 3.53e+07 |
| P78371 | CCT2 | 57.5 | 106 | 7 | 3 | 3 | 3 | 2.62e+07 |
| Q92598 | HSPH1 | 96.8 | 103 | 3 | 2 | 2 | 2 | 1.47e+07 |
| P22626 | HNRNPA2B1 | 37.4 | 102 | 7 | 1 | 2 | 2 |  |
| P62263 | RPS14 | 16.3 | 96 | 16 | 2 | 2 | 2 | 4.63e+07 |
| Q16576 | RBBP7 | 47.8 | 96 | 3 | 1 | 1 | 1 | 9.14e+06 |
| P06454 | PTMA | 12.2 | 96 | 25 | 2 | 2 | 2 | 2.55e+07 |
| Q6IS14 | EIF5AL1 | 16.8 | 92 | 8 | 1 | 1 | 2 | 3.69e+07 |
| Q99832 | CCT7 | 59.3 | 88 | 6 | 2 | 2 | 2 | 6.15e+06 |
| P25398 | RPS12 | 14.5 | 85 | 11 | 1 | 1 | 1 | 3.04e+06 |
| P62753 | RPS6 | 28.7 | 83 | 6 | 1 | 1 | 1 | 1.02e+07 |
| P61313 | RPL15 | 24.1 | 83 | 13 | 2 | 2 | 2 | 1.18e+07 |
| Q04837 | SSBP1 | 17.2 | 81 | 26 | 3 | 3 | 3 | 3.23e+07 |
| P62913 | RPL11 | 20.2 | 81 | 15 | 2 | 2 | 2 | 2.74e+07 |
| P67809 | YBX1 | 35.9 | 79 | 6 | 1 | 1 | 1 | 9.24e+06 |
| P60866 | RPS20 | 13.4 | 78 | 23 | 2 | 2 | 2 | 1.52e+07 |
| P49411 | TUFM | 49.8 | 77 | 4 | 2 | 2 | 2 | 1.70e+07 |
| P08670 | VIM | 53.6 | 76 | 2 | 1 | 1 | 1 | 8.49e+06 |
| P63313 | TMSB10 | 5 | 76 | 50 | 2 | 2 | 2 | 1.55e+07 |
| O00299 | CLIC1 | 26.9 | 74 | 7 | 1 | 1 | 1 | 8.16e+06 |
| P53999 | SUB1 | 14.4 | 70 | 18 | 2 | 2 | 2 | 1.29e+07 |
| Q13162 | PRDX4 | 30.5 | 69 | 8 | 2 | 2 | 2 | 6.82e+07 |
| Q8IX12 | CCAR1 | 132.7 | 68 | 2 | 2 | 2 | 2 | 7.17e+06 |
| O60506 | SYNCRIP | 69.6 | 68 | 2 | 1 | 1 | 1 | 1.11e+07 |
| P62269 | RPS18 | 17.7 | 68 | 7 | 2 | 2 | 2 | 9.07e+07 |
| P29966 | MARCKS | 31.5 | 67 | 6 | 1 | 1 | 1 |  |
| Q8NBS9 | TXNDC5 | 47.6 | 67 | 5 | 1 | 1 | 1 | 5.79e+06 |
| P39019 | RPS19 | 16.1 | 67 | 12 | 2 | 2 | 2 | 2.37e+07 |
| P40429 | RPL13A | 23.6 | 65 | 6 | 1 | 1 | 1 | 5.34e+06 |
| P47914 | RPL29 | 17.7 | 65 | 9 | 1 | 1 | 1 | 1.67e+07 |
| Q9Y285 | FARSA | 57.5 | 62 | 2 | 1 | 1 | 1 | 2.83e+06 |
| Q16777 | H2AC20 | 14 | 62 | 15 | 1 | 1 | 1 | 5.33e+07 |
| P15880 | RPS2 | 31.3 | 61 | 4 | 1 | 1 | 1 | 1.04e+07 |
| P61026 | RAB10 | 22.5 | 58 | 6 | 1 | 1 | 1 | 4.08e+06 |
| P36957 | DLST | 48.7 | 58 | 2 | 1 | 1 | 2 | 1.06e+08 |
| P00505 | GOT2 | 47.5 | 56 | 3 | 1 | 1 | 1 | 6.97e+06 |
| P61158 | ACTR3 | 47.3 | 56 | 3 | 1 | 1 | 1 | 2.48e+06 |
| P62750 | RPL23A | 17.7 | 56 | 8 | 1 | 1 | 1 | 4.00e+07 |
| Q15102 | PAFAH1B3 | 25.7 | 56 | 4 | 1 | 1 | 1 | 3.35e+06 |
| P30040 | ERP29 | 29 | 56 | 4 | 1 | 1 | 1 | 8.05e+06 |
| P23378 | GLDC | 112.7 | 55 | 1 | 1 | 1 | 1 | 1.06e+07 |
| P23921 | RRM1 | 90 | 55 | 2 | 1 | 1 | 1 | 1.79e+06 |
| P62906 | RPL10A | 24.8 | 54 | 4 | 1 | 1 | 1 | 3.60e+07 |
| P55060 | CSE1L | 110.3 | 53 | 1 | 1 | 1 | 1 | 1.12e+08 |
| P05198 | EIF2S1 | 36.1 | 53 | 6 | 1 | 1 | 1 | 3.47e+06 |
| P51665 | PSMD7 | 37 | 53 | 5 | 1 | 1 | 1 | 3.45e+06 |
| P26639 | TARS1 | 83.4 | 52 | 1 | 1 | 1 | 1 | 7.58e+06 |
| P31040 | SDHA | 72.6 | 51 | 2 | 1 | 1 | 1 |  |
| Q9NYF8 | BCLAF1 | 106.1 | 51 | 1 | 1 | 1 | 1 | 5.83e+06 |
| P62280 | RPS11 | 18.4 | 50 | 6 | 1 | 1 | 1 | 2.03e+07 |
| P35637 | FUS | 53.4 | 50 | 3 | 1 | 1 | 1 | 3.96e+06 |
| P57088 | TMEM33 | 28 | 48 | 5 | 1 | 1 | 1 | 5.75e+06 |
| O00264 | PGRMC1 | 21.7 | 48 | 7 | 1 | 1 | 1 | 7.21e+06 |
| P62899 | RPL31 | 14.5 | 48 | 7 | 1 | 1 | 1 | 4.51e+07 |
| P02545 | LMNA | 74.1 | 47 | 2 | 1 | 1 | 1 | 2.74e+06 |
| Q9P2E9 | RRBP1 | 152.4 | 47 | 0 | 1 | 1 | 1 | 3.68e+07 |
| P04844 | RPN2 | 69.2 | 47 | 3 | 1 | 1 | 1 |  |
| Q9NQH7 | XPNPEP3 | 57 | 47 | 1 | 1 | 1 | 1 | 9.72e+07 |
| P23284 | PPIB | 23.7 | 46 | 4 | 1 | 1 | 1 | 2.73e+07 |
| P00966 | ASS1 | 46.5 | 46 | 2 | 1 | 1 | 1 | 3.73e+07 |
| P46781 | RPS9 | 22.6 | 46 | 4 | 1 | 1 | 1 | 1.09e+07 |
| P30044 | PRDX5 | 22.1 | 45 | 4 | 1 | 1 | 1 | 5.82e+06 |
| Q15185 | PTGES3 | 18.7 | 45 | 8 | 1 | 1 | 1 | 6.08e+06 |
| O60812 | HNRNPCL1 | 32.1 | 45 | 4 | 1 | 1 | 1 | 7.93e+06 |
| Q99729 | HNRNPAB | 36.2 | 45 | 4 | 1 | 1 | 1 | 1.92e+06 |
| O15479 | MAGEB2 | 35.3 | 43 | 5 | 1 | 1 | 1 | 2.10e+06 |
| P07814 | EPRS1 | 170.5 | 43 | 1 | 1 | 1 | 1 | 3.02e+06 |
| P32969 | RPL9P9 | 21.9 | 43 | 6 | 1 | 1 | 1 | 4.10e+06 |
| Q02978 | SLC25A11 | 34 | 43 | 5 | 1 | 1 | 1 | 1.88e+06 |
| P12955 | PEPD | 54.5 | 42 | 2 | 1 | 1 | 1 | 8.97e+05 |
| Q9HB71 | CACYBP | 26.2 | 42 | 6 | 1 | 1 | 1 | 5.13e+06 |
| P00390 | GSR | 56.2 | 41 | 2 | 1 | 1 | 1 | 4.58e+06 |
| P39656 | DDOST | 50.8 | 40 | 2 | 1 | 1 | 1 | 3.30e+06 |
| Q13838 | DDX39B | 49 | 39 | 3 | 1 | 1 | 1 | 2.65e+06 |

* The table displays proteins exclusively found in the UCHL3 group, absent in the vector group, or those significantly elevated in the UCHL3 group while markedly reduced in the vector group.
