# Supplementary material for: UCHL3 depletion inhibits gastric cancer progression and enhances palbociclib sensitivity by regulating the AKT/CCND1 signaling axis via ENO1 ubiquitination
Source: Cell Death Dis. 2025 Nov 21;16(1):850. doi: 10.1038/s41419-025-08153-3 (PMC12638999; doi:10.1038/s41419-025-08153-3)
Supplement: Supplementary file 2 — Supplemental Material-Raw data [file 41419_2025_8153_MOESM2_ESM.pdf]

Supplemental Material-Raw data

Figure 1H-I

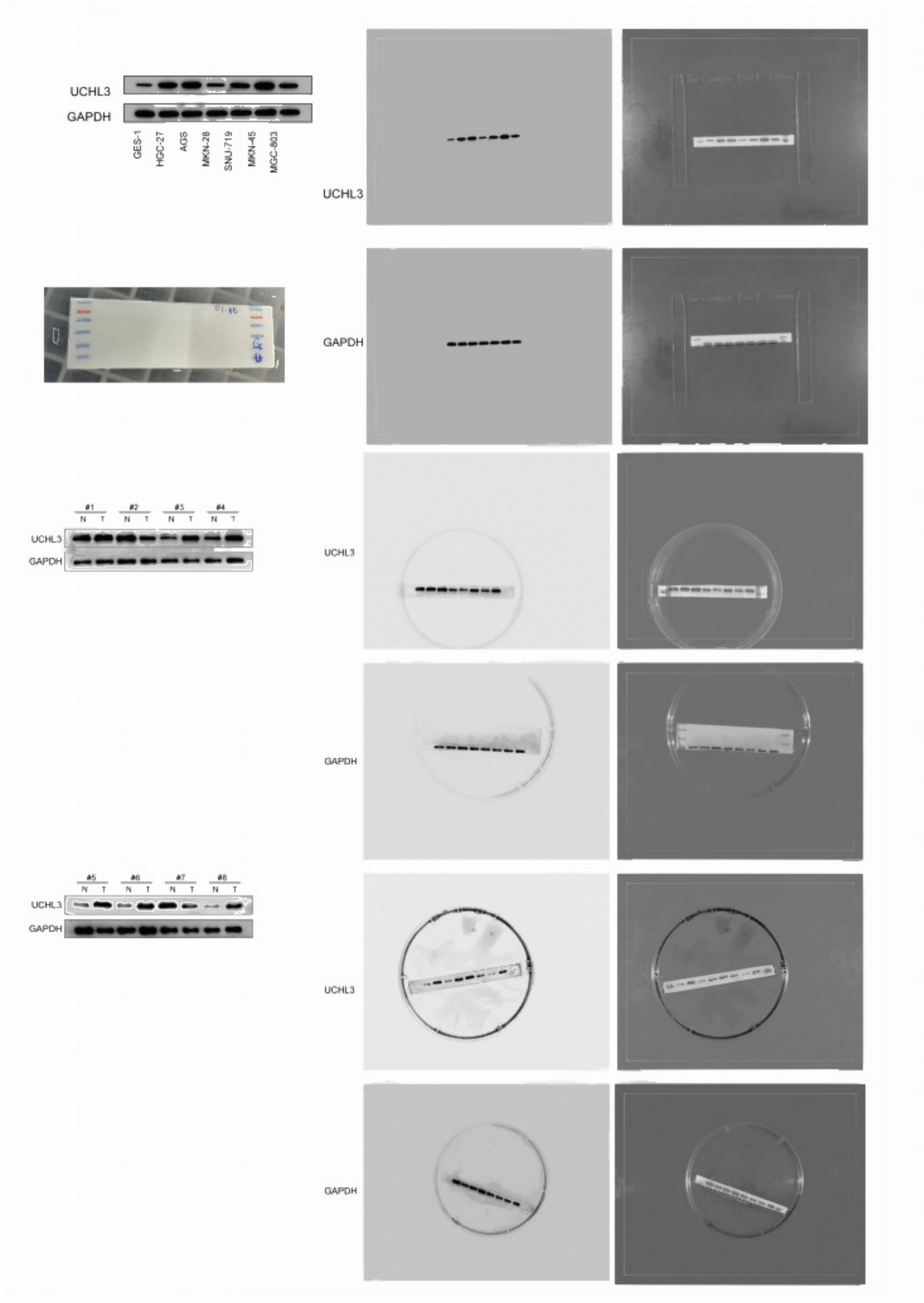

Figure 2

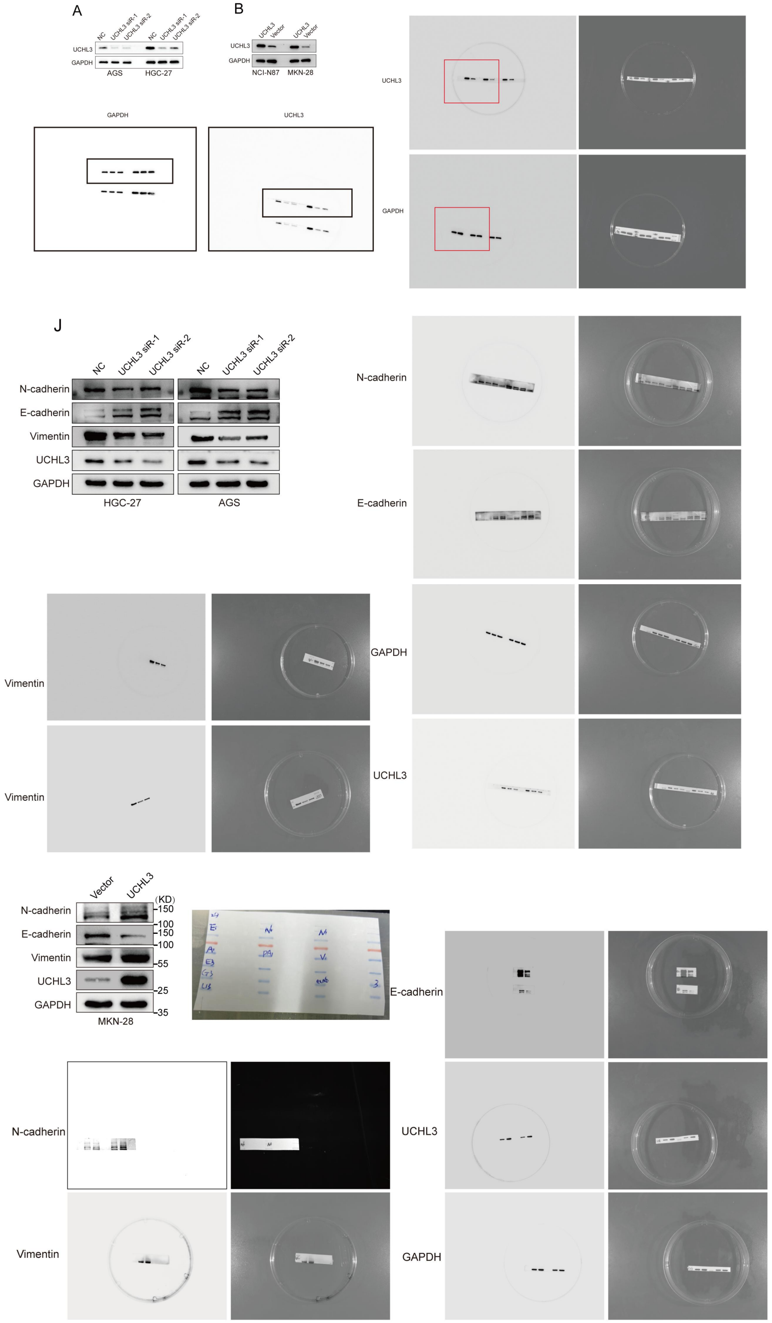
A
NC
UCHL3 siR-1
UCHL3 siR-2
UCHL3
GAPDH
AGS
HGC-27
B
UCHL3
Vector
NCI-N87
MKN-28
GAPDH
UCHL3
J
N-cadherin
E-cadherin
Vimentin
UCHL3
GAPDH
HGC-27
AGS
Vimentin
Vimentin
N-cadherin
E-cadherin
GAPDH
UCHL3
Vector
UCHL3
(KD)
150
100
150
100
55
25
35
MKN-28
E-cadherin
UCHL3
N-cadherin
Vimentin
GAPDH

Figure 4

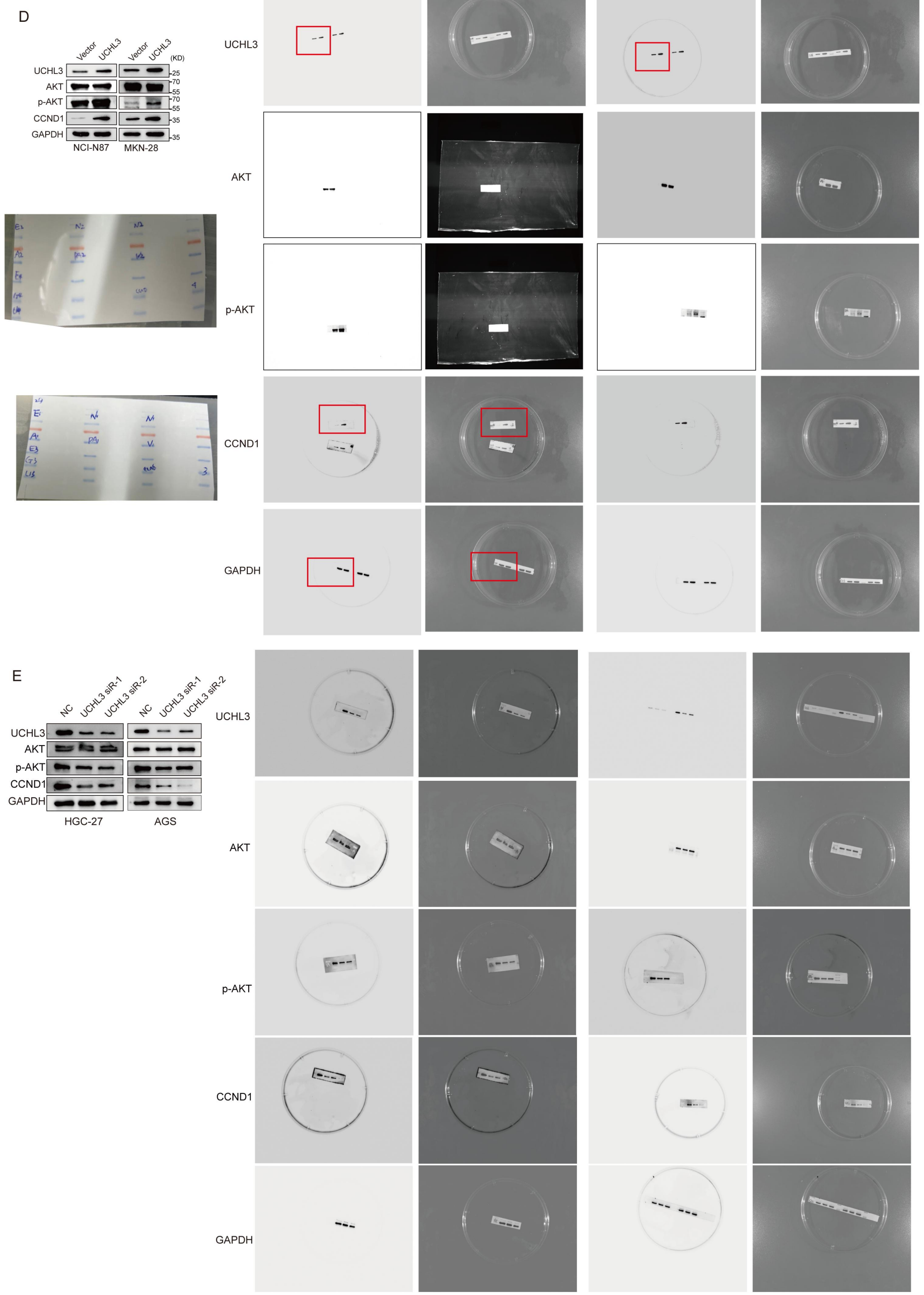
D
Vector
UCHL3
Vector
UCHL3
(KD)
UCHL3
AKT
p-AKT
CCND1
GAPDH
-25
-70
-55
-70
-55
-35
-35
NCI-N87
MKN-28
UCHL3
AKT
p-AKT
CCND1
GAPDH
E
NC
UCHL3 siR-1
UCHL3 siR-2
NC
UCHL3 siR-1
UCHL3 siR-2
UCHL3
AKT
p-AKT
CCND1
GAPDH
HGC-27
AGS
UCHL3
AKT
p-AKT
CCND1
GAPDH

Figure 4

H

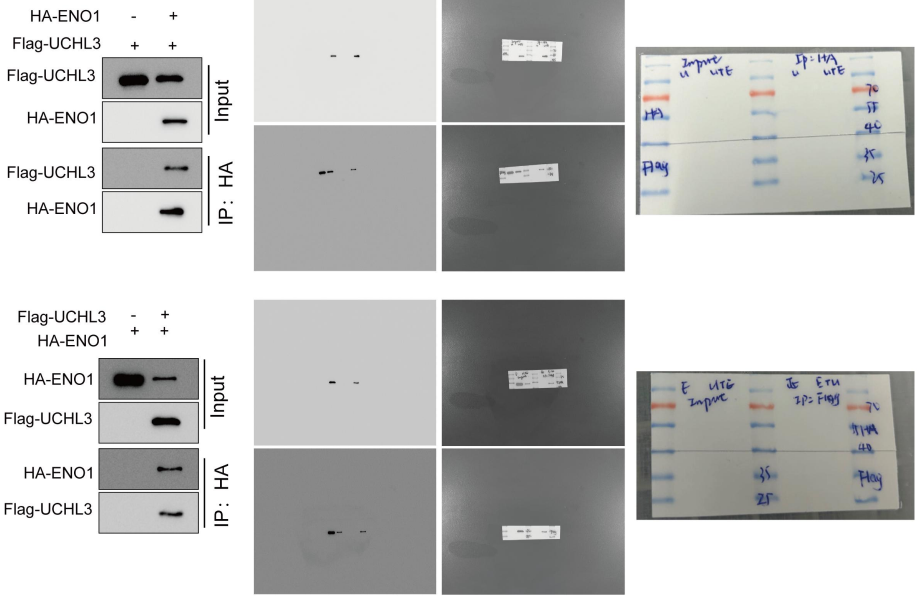
HA-ENO1 - +
Flag-UCHL3 + +
Flag-UCHL3
HA-ENO1
Input
Flag-UCHL3
HA-ENO1
IP: HA
Flag-UCHL3 - +
HA-ENO1 + +
HA-ENO1
Flag-UCHL3
Input
HA-ENO1
Flag-UCHL3
IP: HA

I

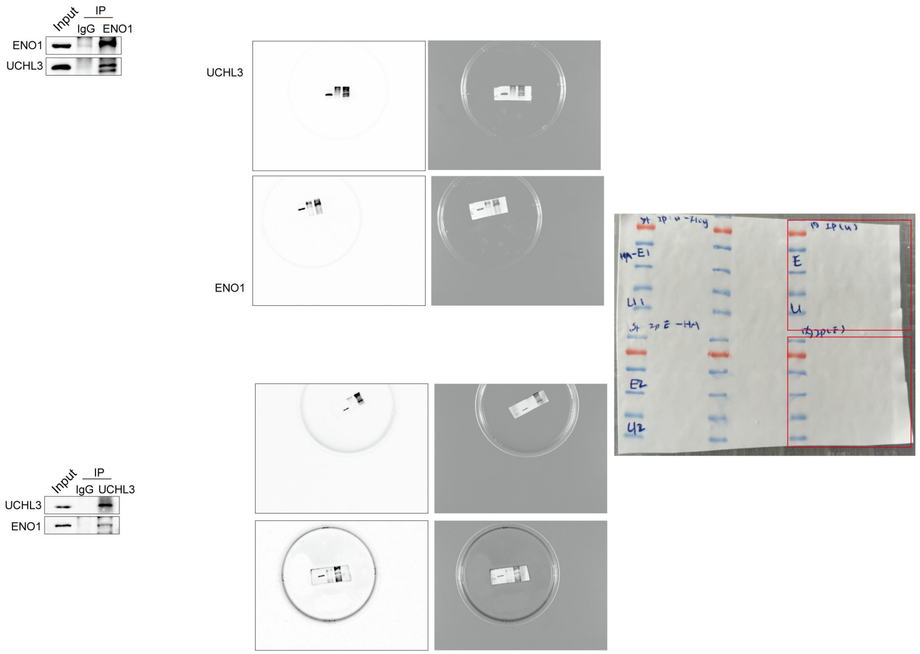
Input
IP
IgG
ENO1
ENO1
UCHL3
UCHL3
ENO1
Input
IP
IgG
UCHL3
UCHL3
ENO1

Figure 4

L

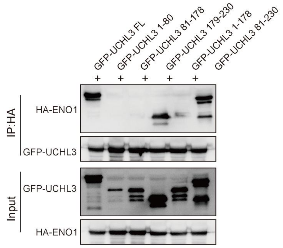
GFP-UCHL3 FL
GFP-UCHL3 1-80
GFP-UCHL3 81-178
GFP-UCHL3 179-230
GFP-UCHL3 1-178
GFP-UCHL3 81-230
+ + + + + +
IP:HA
HA-ENO1
GFP-UCHL3
Input
GFP-UCHL3
HA-ENO1

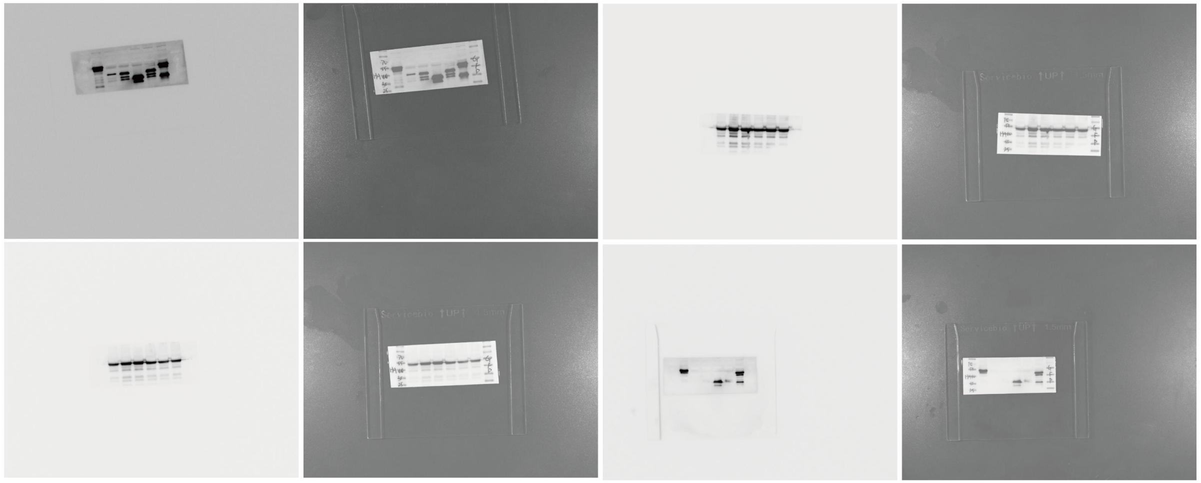

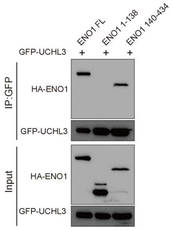
ENO1 FL
ENO1 1-138
ENO1 140-434
GFP-UCHL3
+ + +
IP:GFP
HA-ENO1
GFP-UCHL3
Input
HA-ENO1
GFP-UCHL3

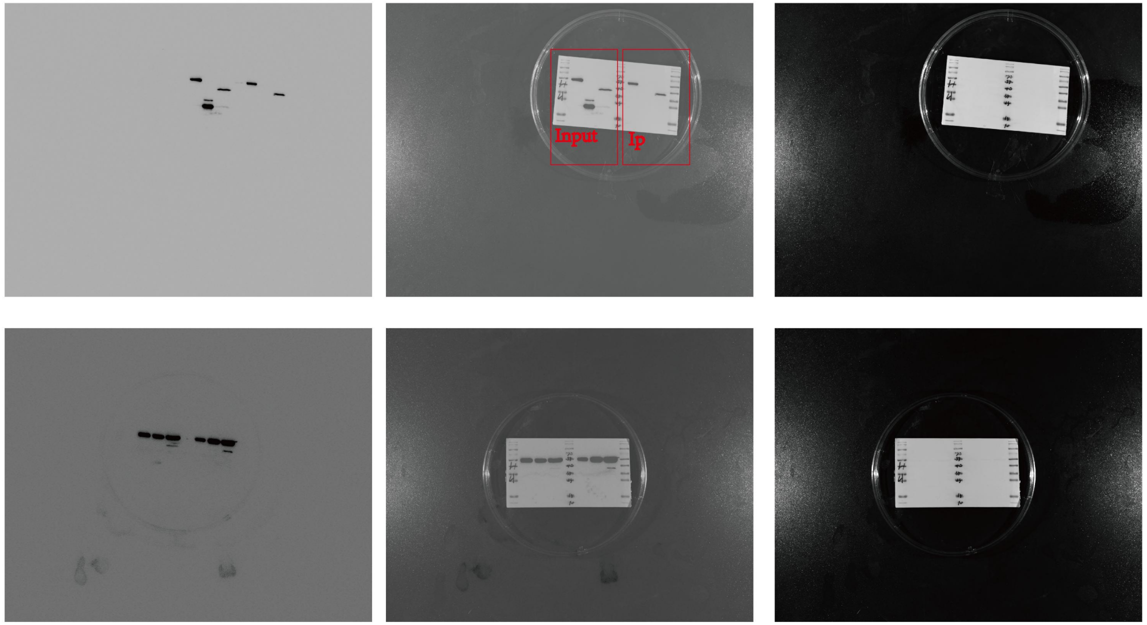
Input
Ip

Figure 5

A

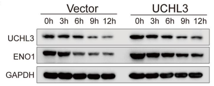

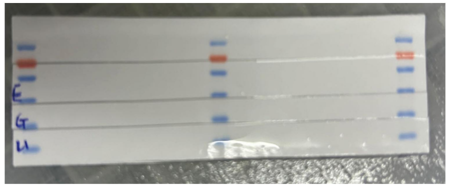

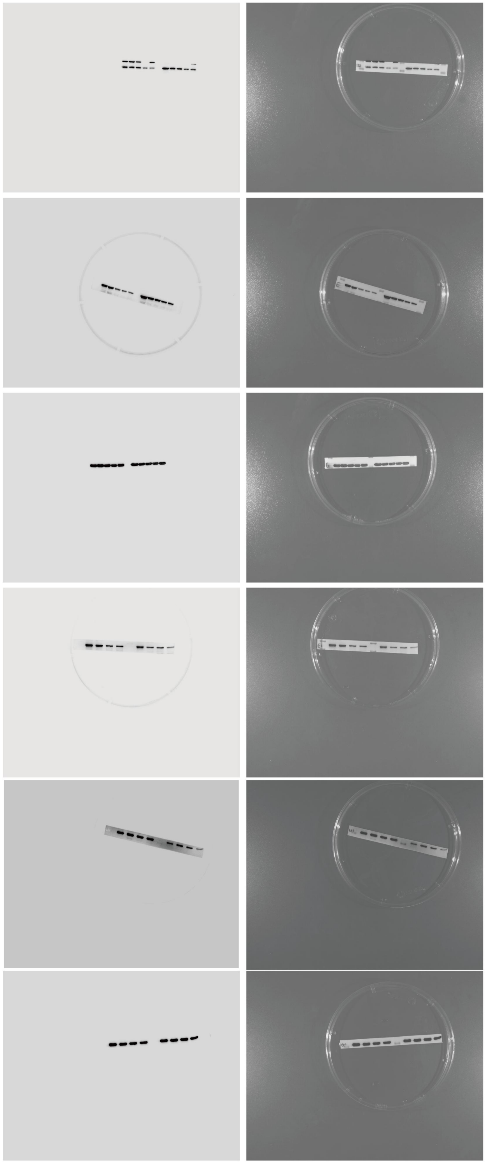

UCHL3

ENO1

GAPDH

ENO1

UCHL3

GAPDH

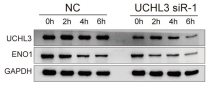

Figure 5

B

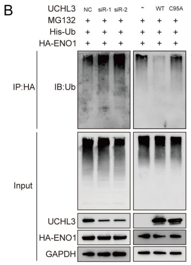
UCHL3 NC siR-1 siR-2 - WT C95A
MG132
His-Ub
HA-ENO1
IP:HA
IB:Ub
Input
UCHL3
HA-ENO1
GAPDH

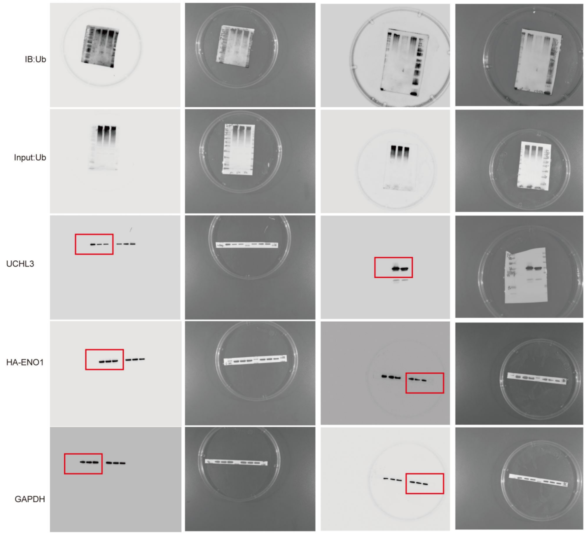
IB:Ub
Input:Ub
UCHL3
HA-ENO1
GAPDH

C

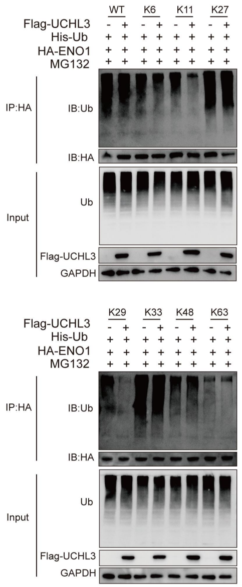
WT K6 K11 K27
Flag-UCHL3
His-Ub
HA-ENO1
MG132
IP:HA
IB:Ub
IB:HA
Input
Ub
Flag-UCHL3
GAPDH
K29 K33 K48 K63
Flag-UCHL3
His-Ub
HA-ENO1
MG132
IP:HA
IB:Ub
IB:HA
Input
Ub
Flag-UCHL3
GAPDH

F

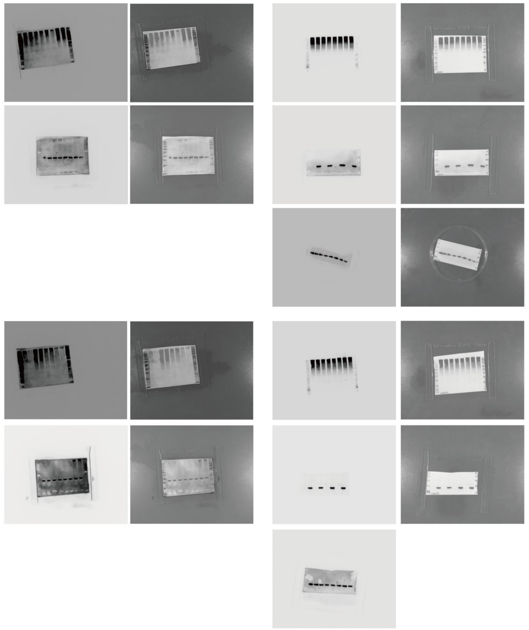

Figure 5

B

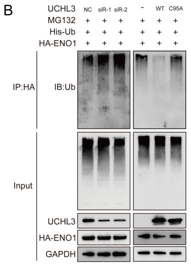
UCHL3
MG132
His-Ub
HA-ENO1
NC
siR-1
siR-2
-
WT
C95A
IP:HA
IB:Ub
Input
UCHL3
HA-ENO1
GAPDH

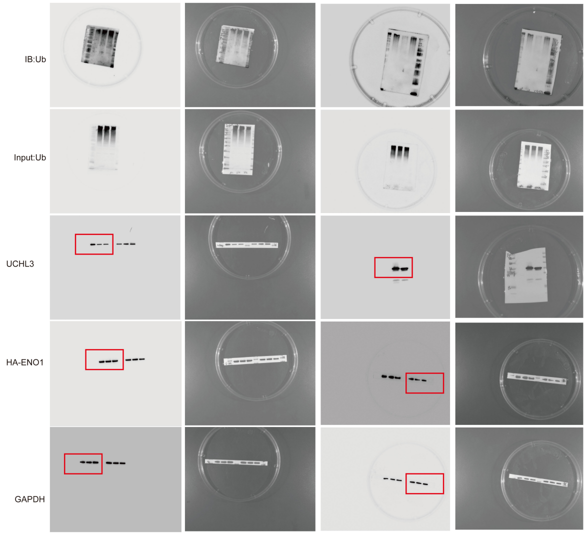
IB:Ub
Input:Ub
UCHL3
HA-ENO1
GAPDH

C

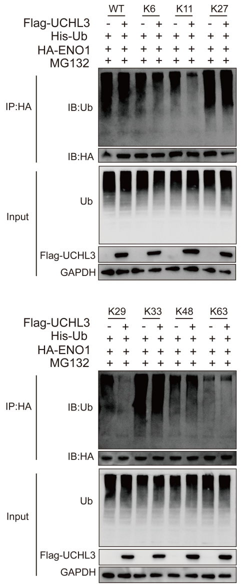
WT
K6
K11
K27
K29
K33
K48
K63
Flag-UCHL3
His-Ub
HA-ENO1
MG132
IP:HA
IB:Ub
IB:HA
Input
Ub
Flag-UCHL3
GAPDH

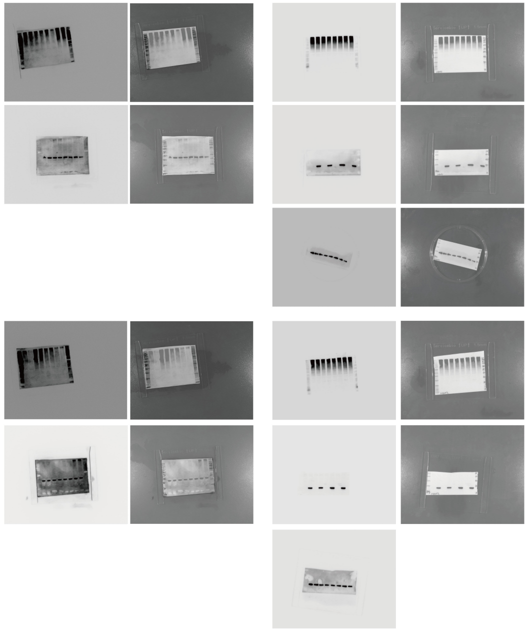

Figure 6

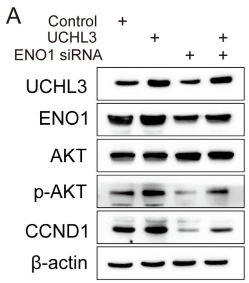
A
Control
UCHL3
ENO1 siRNA
UCHL3
ENO1
AKT
p-AKT
CCND1
β-actin

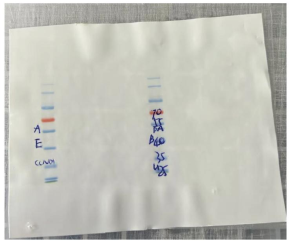

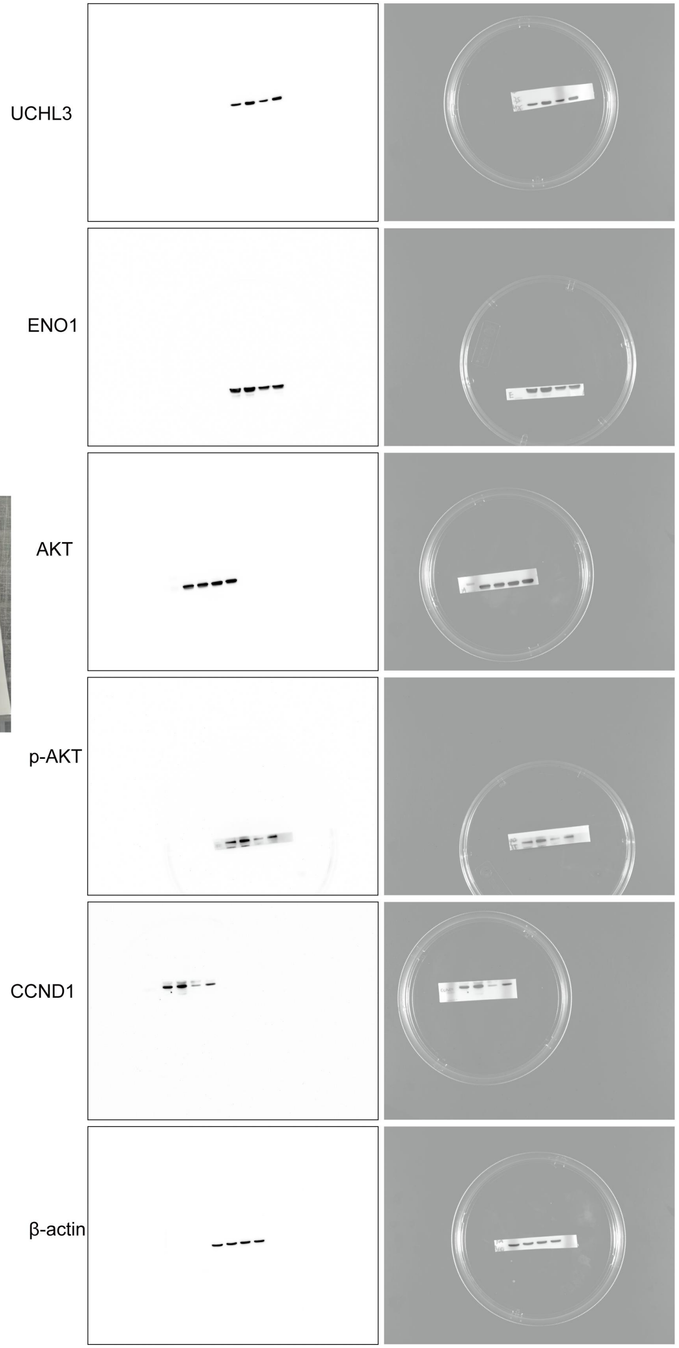
UCHL3
ENO1
AKT
p-AKT
CCND1
β-actin

Figure 7

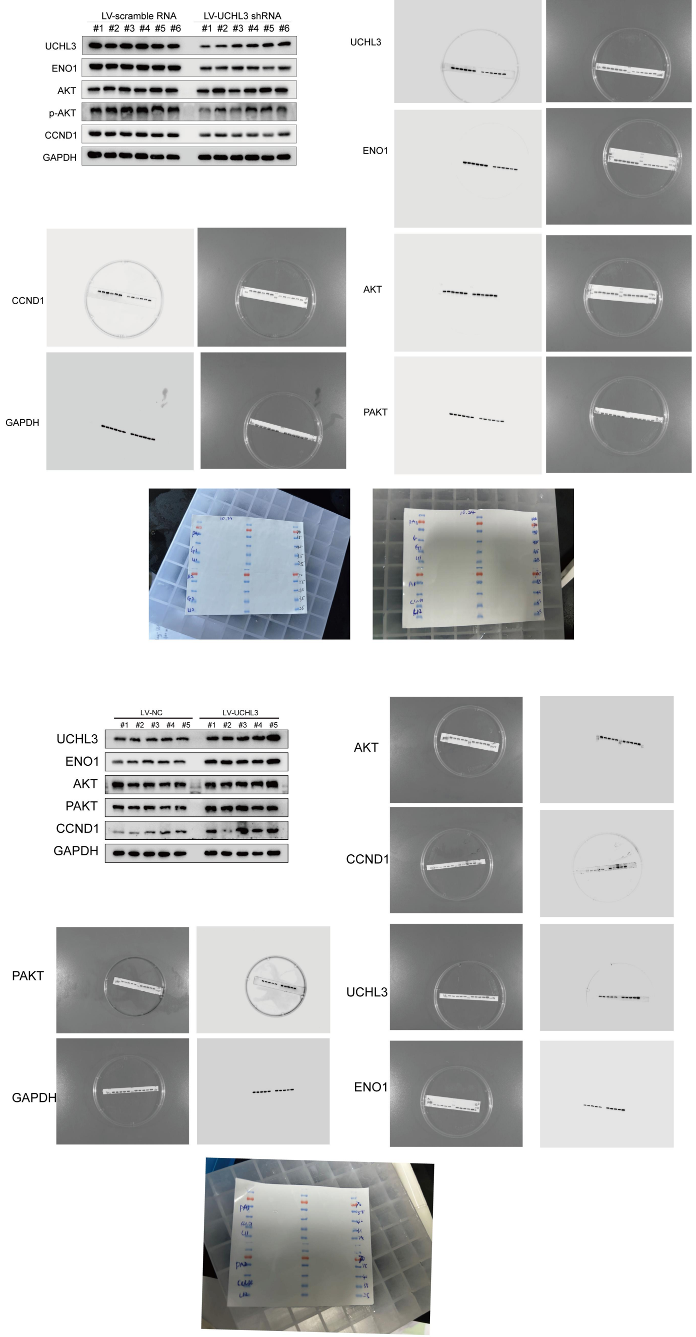
LV-scramble RNA
LV-UCHL3 shRNA
#1 #2 #3 #4 #5 #6 #1 #2 #3 #4 #5 #6
UCHL3
ENO1
AKT
p-AKT
CCND1
GAPDH
UCHL3
ENO1
AKT
PAKT
CCND1
GAPDH
LV-NC
LV-UCHL3
#1 #2 #3 #4 #5 #1 #2 #3 #4 #5
UCHL3
ENO1
AKT
PAKT
CCND1
GAPDH
AKT
CCND1
UCHL3
ENO1
PAKT
GAPDH

Figure 8

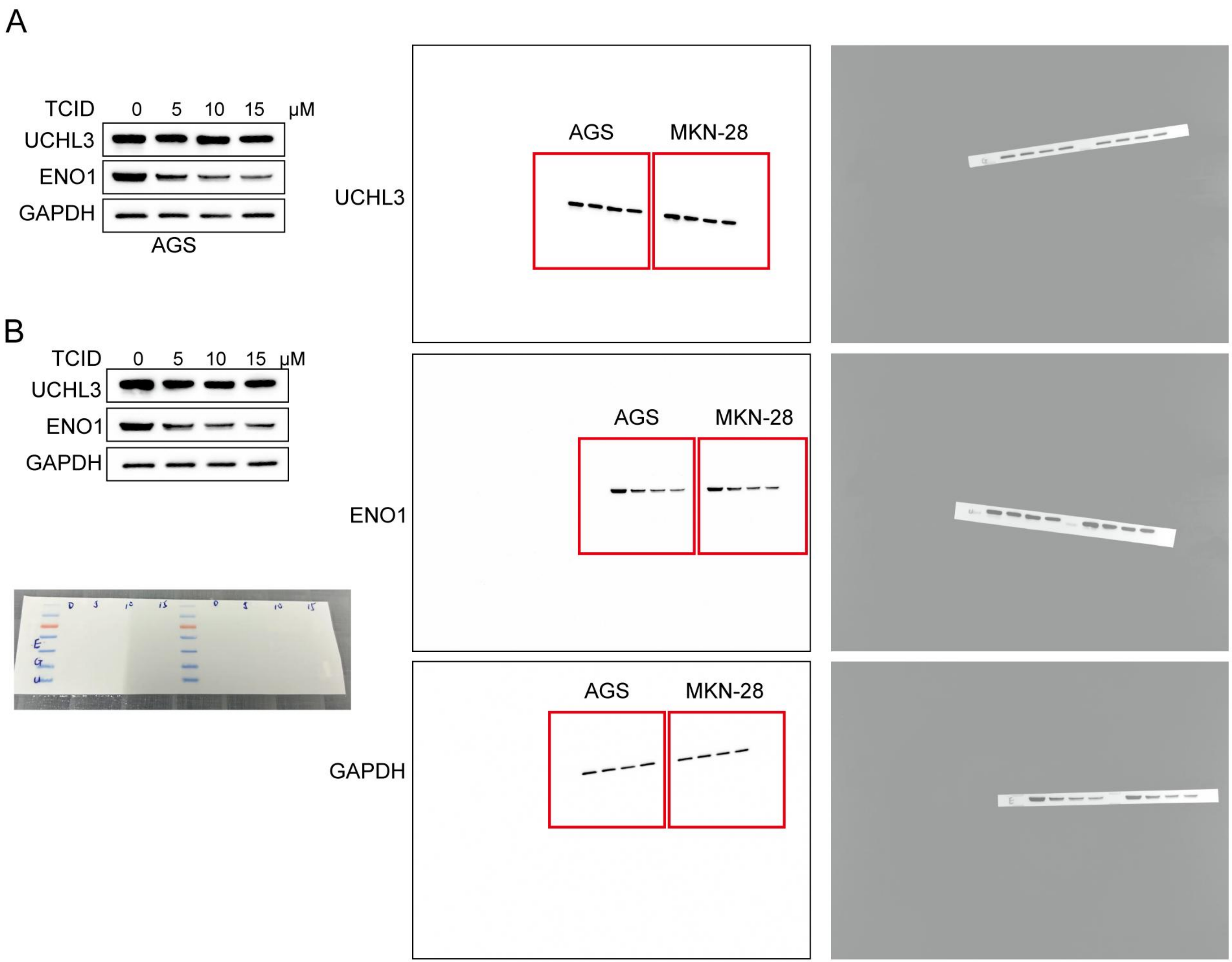
A
TCID 0 5 10 15 μM
UCHL3
ENO1
GAPDH
AGS
B
TCID 0 5 10 15 μM
UCHL3
ENO1
GAPDH
UCHL3
AGS
MKN-28
ENO1
AGS
MKN-28
GAPDH
AGS
MKN-28

Figure 8

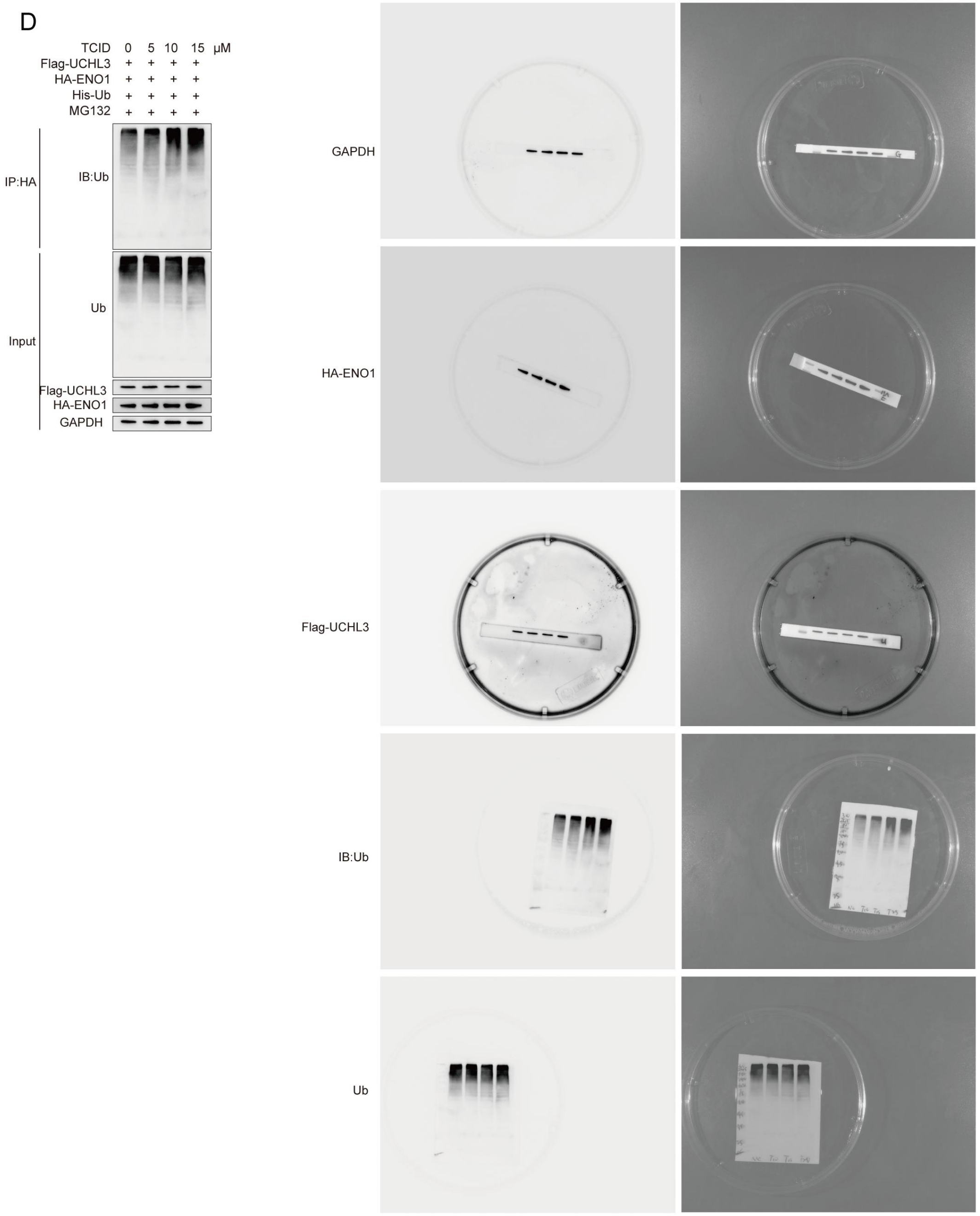
D
TCID 0 5 10 15 μM
Flag-UCHL3 + + + +
HA-ENO1 + + + +
His-Ub + + + +
MG132 + + + +
IP:HA
IB:Ub
Input
Ub
Flag-UCHL3
HA-ENO1
GAPDH
GAPDH
HA-ENO1
Flag-UCHL3
IB:Ub
Ub

Fig. S2

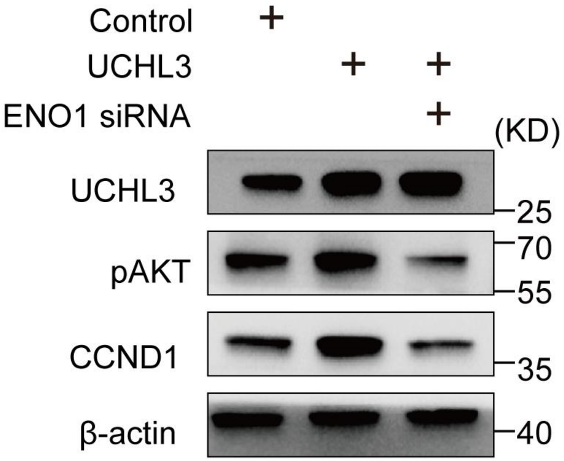
Control
UCHL3
ENO1 siRNA
(KD)
UCHL3
25
pAKT
70
55
CCND1
35
β-actin
40

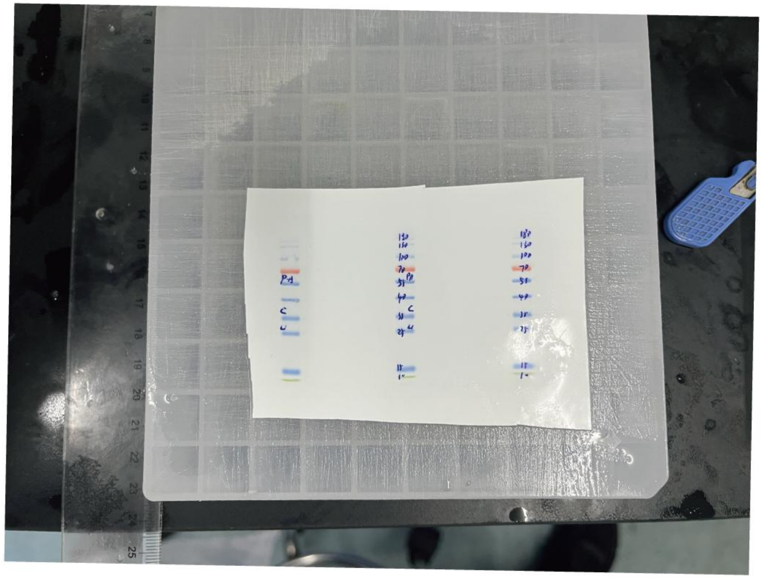

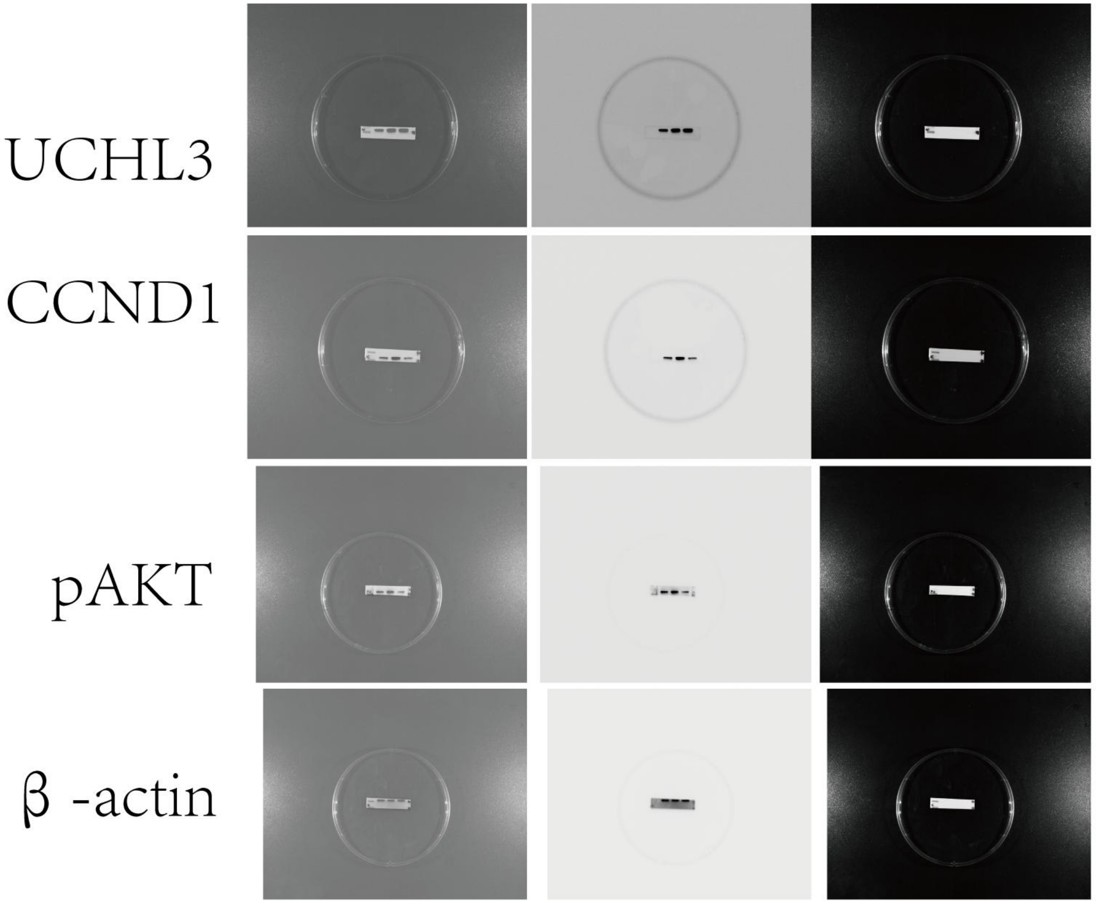
UCHL3
CCND1
pAKT
β -actin

Fig.S3

A

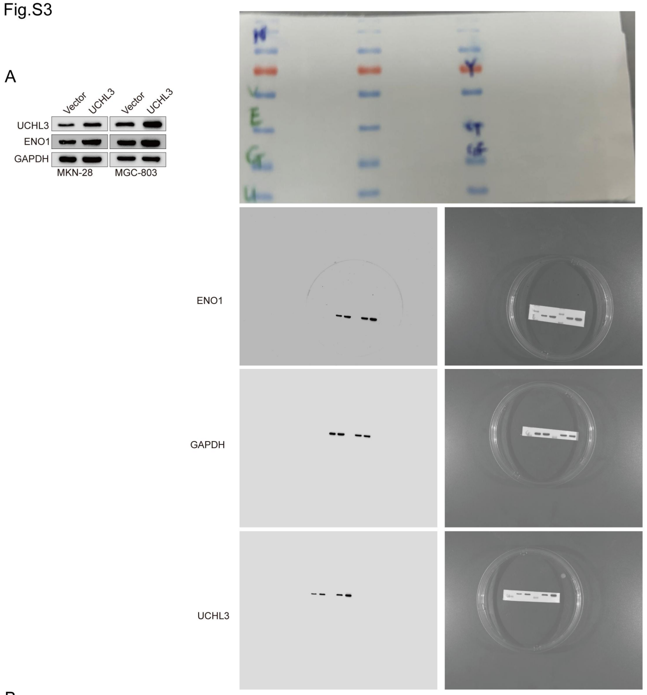
Vector
UCHL3
Vector
UCHL3
UCHL3
ENO1
GAPDH
MKN-28
MGC-803
ENO1
GAPDH
UCHL3

B

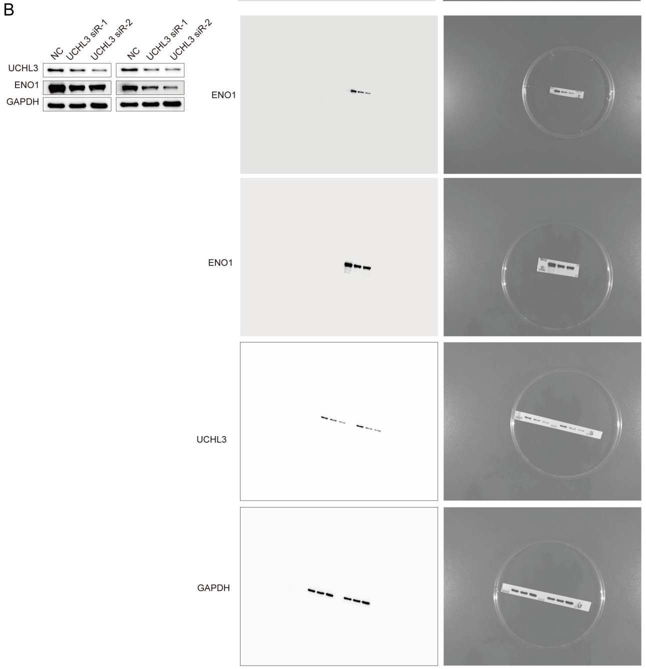
NC
UCHL3 siR-1
UCHL3 siR-2
NC
UCHL3 siR-1
UCHL3 siR-2
UCHL3
ENO1
GAPDH
ENO1
ENO1
UCHL3
GAPDH

Fig.S3

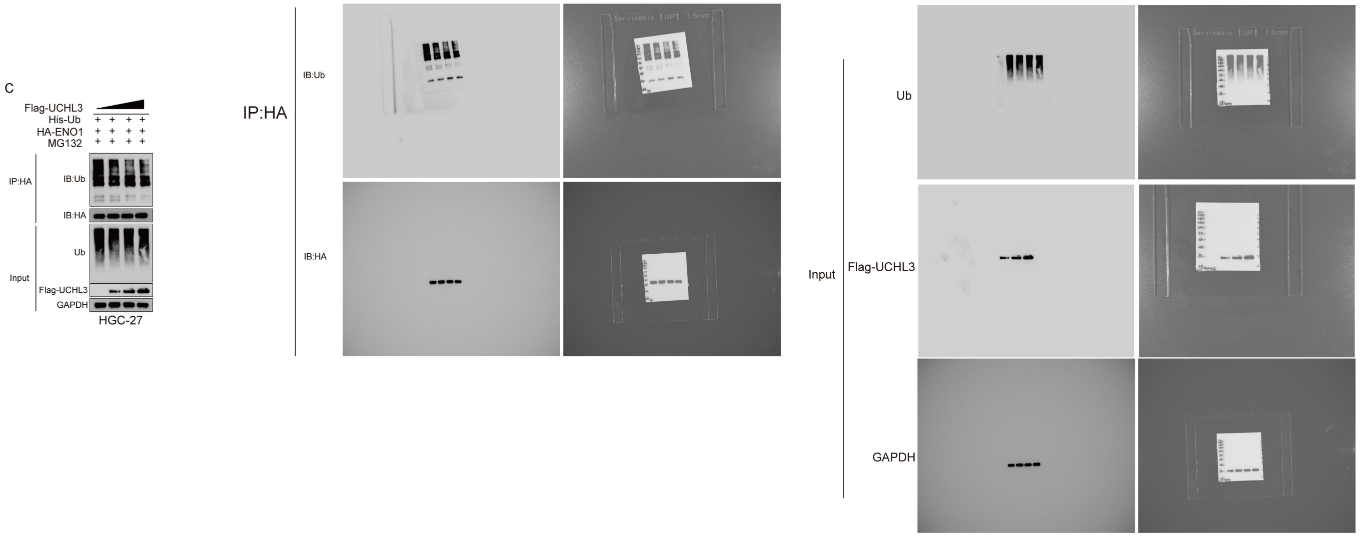
C
Flag-UCHL3
His-Ub
HA-ENO1
MG132
IP:HA
IB:Ub
IB:HA
Input
Ub
Flag-UCHL3
GAPDH
HGC-27
IP:HA
IB:Ub
IB:HA
Input
Ub
Flag-UCHL3
GAPDH

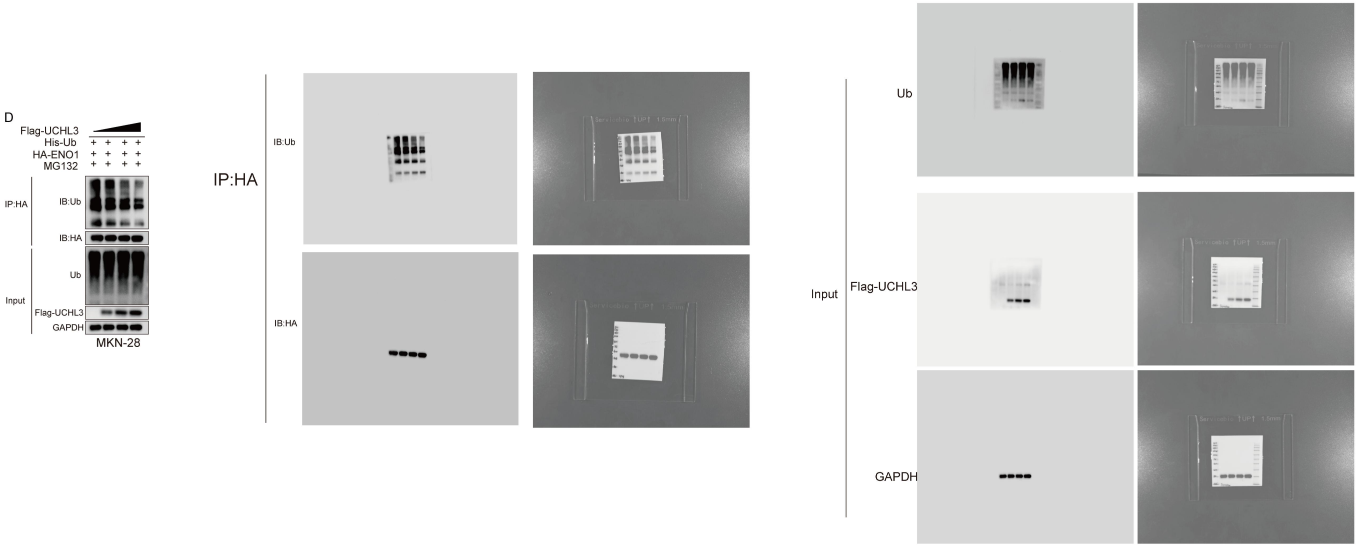
D
Flag-UCHL3
His-Ub
HA-ENO1
MG132
IP:HA
IB:Ub
IB:HA
Input
Ub
Flag-UCHL3
GAPDH
MKN-28
IP:HA
IB:Ub
IB:HA
Input
Ub
Flag-UCHL3
GAPDH

Fig.S3

E

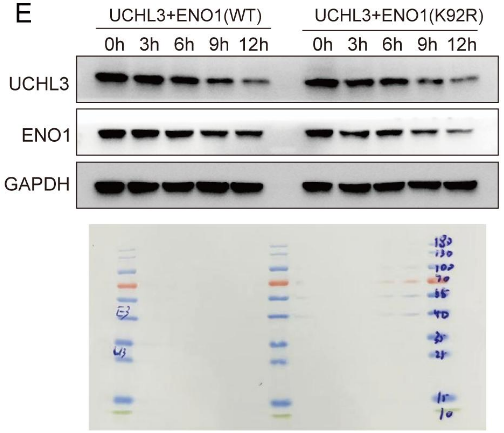
UCHL3+ENO1(WT)
UCHL3+ENO1(K92R)
0h 3h 6h 9h 12h
0h 3h 6h 9h 12h
UCHL3
ENO1
GAPDH

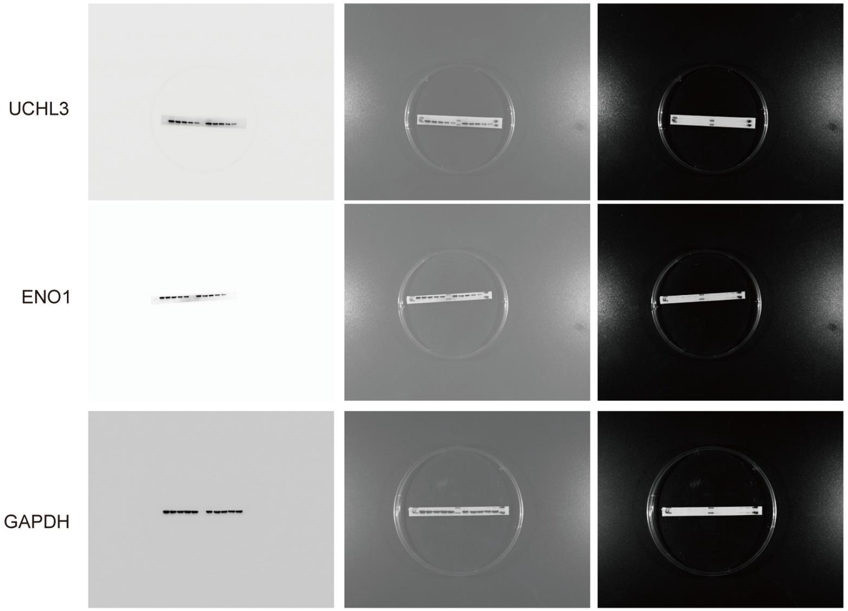
UCHL3
ENO1
GAPDH

F

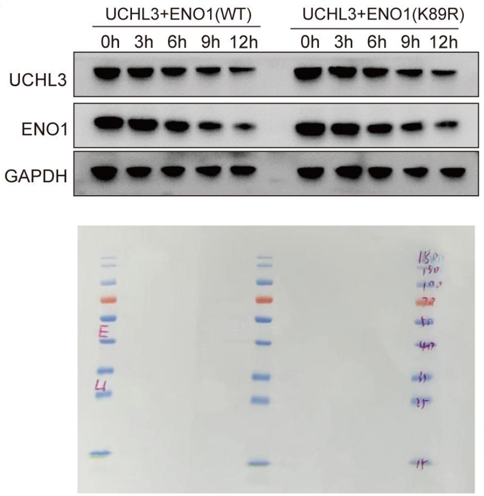
UCHL3+ENO1(WT)
UCHL3+ENO1(K89R)
0h 3h 6h 9h 12h
0h 3h 6h 9h 12h
UCHL3
ENO1
GAPDH

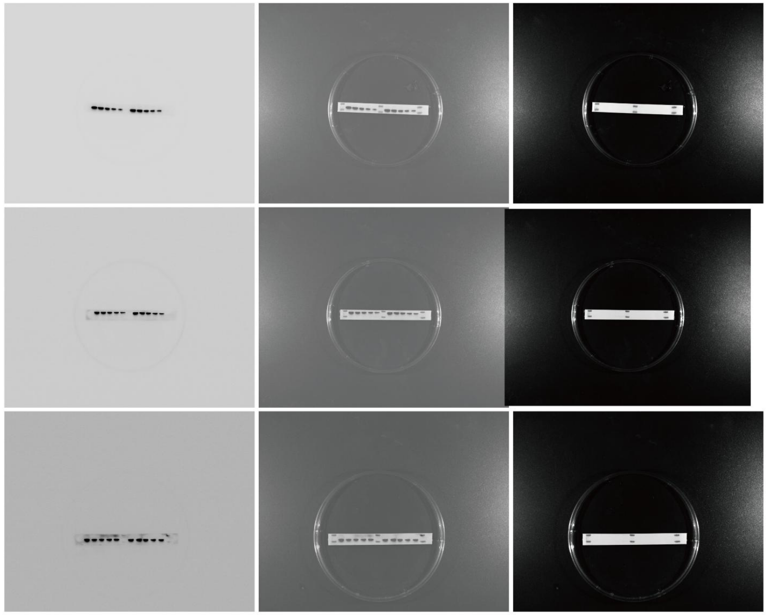

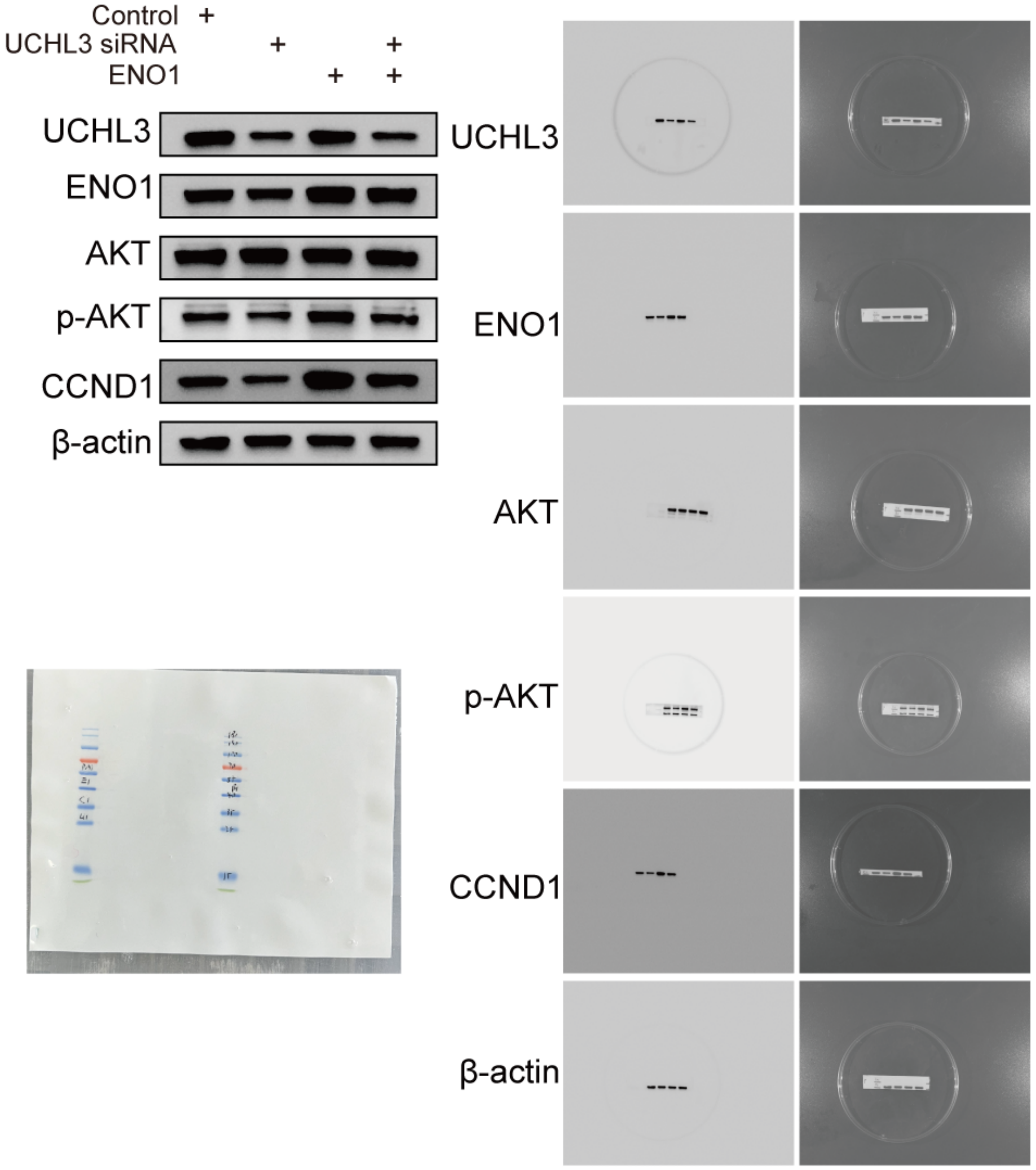
Control
UCHL3 siRNA
ENO1
UCHL3
ENO1
AKT
p-AKT
CCND1
β-actin
UCHL3
ENO1
AKT
p-AKT
CCND1
β-actin
